# Macrophage maturation from blood monocytes is altered in people with HIV, and is linked to serum lipid profiles and activation indices: A model for studying atherogenic mechanisms

**DOI:** 10.1371/journal.ppat.1008869

**Published:** 2020-10-01

**Authors:** Emily R. Bowman, Cheryl M. Cameron, Brian Richardson, Manjusha Kulkarni, Janelle Gabriel, Morgan J. Cichon, Kenneth M. Riedl, Yousef Mustafa, Michael Cartwright, Brandon Snyder, Subha V. Raman, David A. Zidar, Susan L. Koletar, Martin P. Playford, Nehal N. Mehta, Scott F. Sieg, Michael L. Freeman, Michael M. Lederman, Mark J. Cameron, Nicholas T. Funderburg

**Affiliations:** 1 School of Health and Rehabilitation Sciences, Division of Medical Laboratory Science, Ohio State University, Columbus, Ohio, United States of America; 2 Department of Nutrition, Case Western Reserve University, Cleveland, Ohio, United States of America; 3 Department of Population and Quantitative Health Sciences, Case Western Reserve University, Cleveland, Ohio, United States of America; 4 Department of Food Science & Technology and the Nutrient & Phytochemical Shared Resource, Comprehensive Cancer Center, Ohio State University, Columbus, Ohio, United States of America; 5 Department of Internal Medicine, Division of Cardiovascular Medicine, Ohio State University, Columbus, Ohio, United States of America; 6 Harrington Heart and Vascular Institute, University Hospitals Cleveland Medical Center, Cleveland, Ohio, United States of America; 7 Department of Medicine, Division of Infectious Diseases, Ohio State University, Columbus, Ohio, United States of America; 8 Section of Inflammation and Cardiometabolic Diseases, National Heart, Lung, and Blood Institute, National Institutes of Health, Bethesda, MD, United States of America; 9 Department of Medicine, Division of Infectious Diseases and HIV Medicine, Case Western Reserve University/University Hospitals of Cleveland, Cleveland, Ohio, United States of America; Vaccine Research Center, UNITED STATES

## Abstract

People with HIV (PWH) are at increased risk for atherosclerotic cardiovascular disease (ASCVD). Proportions of vascular homing monocytes are enriched in PWH; however, little is known regarding monocyte-derived macrophages (MDMs) that may drive atherosclerosis in this population. We isolated PBMCs from people with and without HIV, and cultured these cells for 5 days in medium containing autologous serum to generate MDMs. Differential gene expression (DGE) analysis of MDMs from PWH identified broad alterations in innate immune signaling (IL-1β, TLR expression, PPAR βδ) and lipid processing (LXR/RXR, ACPP, SREBP1). Transcriptional changes aligned with the functional capabilities of these cells. Expression of activation markers and innate immune receptors (CD163, TLR4, and CD300e) was altered on MDMs from PWH, and these cells produced more TNFα, reactive oxygen species (ROS), and matrix metalloproteinases (MMPs) than did cells from people without HIV. MDMs from PWH also had greater lipid accumulation and uptake of oxidized LDL. PWH had increased serum levels of free fatty acids (FFAs) and ceramides, with enrichment of saturated FAs and a reduction in polyunsaturated FAs. Levels of lipid classes and species that are associated with CVD correlated with unique DGE signatures and altered metabolic pathway activation in MDMs from PWH. Here, we show that MDMs from PWH display a pro-atherogenic phenotype; they readily form foam cells, have altered transcriptional profiles, and produce mediators that likely contribute to accelerated ASCVD.

## Author summary

People with HIV (PWH) are at greater risk for developing cardiovascular disease (CVD) than the general public, but the mechanisms underlying this increased risk are poorly understood. Macrophages play key roles in the pathogenesis of atherosclerosis, and are potential targets for therapeutic intervention. Here, we investigate phenotypic and functional abnormalities in monocyte-derived macrophages (MDMs) isolated from PWH that may drive CVD risk in this population. MDMs were differentiated in the presence of autologous serum, enabling us to explore the contributions of serum components (lipids, inflammatory cytokines, microbial products) as drivers of altered MDM function. We link serum levels of inflammatory biomarkers and CVD-associated lipid species to MDM activation. Our study provides new insight into drivers of pro-atherogenic MDM phenotype in PWH, and identifies directions for future study and potential intervention strategies to mitigate CVD risk.

## Introduction

Human immunodeficiency virus (HIV) infection and antiretroviral therapy (ART) use are linked to an increased incidence of atherosclerotic cardiovascular disease (ASCVD) [1–3]. Immune activation persists in ART-treated people with HIV (PWH), and markers of immune activation (i.e. IL-6, C-reactive protein) are predictive of mortality in PWH [4–7]. Inflammation is also central to the development of atherosclerosis in the general population [8, 9]. Markers of myeloid cell activation, including soluble CD14 (sCD14) and sCD163, are elevated in PWH and have been linked to carotid artery atheroma development [10, 11]. Levels of sCD14 [12] are also associated with CVD risk in individuals without HIV, suggesting that regardless of HIV serostatus, monocytes contribute to CVD progression. While levels of sCD14 and sCD163 may be predictive of CVD events, they provide little insight into the specific mechanisms underlying the development of ACVD. Identifying myeloid cell activation and functional profiles that are associated with elevated plasma levels of these biomarkers may help to define how monocytes/macrophages contribute to accelerated ASCVD in PWH.

Circulating monocytes, including inflammatory (CD14^+^CD16^+^) and patrolling (CD14^dim^CD16^+^) subsets, express high levels of vascular adhesion molecules. CD16+ monocytes are enriched in PWH and in individuals without HIV who have recently had an acute coronary event [13]. Monocytes migrate into the subendothelial space and differentiate into macrophages [14–19]; these cells produce several molecules that contribute to atherosclerosis, including inflammatory cytokines, reactive oxygen species (ROS), and matrix metalloproteinases (MMPs). Macrophages can also become lipid laden foam cells due to dysregulated lipid uptake, metabolism, and efflux. Macrophages recovered from atherosclerotic plaques have a decreased ability to migrate and do not effectively mediate resolution of inflammation [20, 21]. These cells also contribute to necrotic core formation and plaque instability due to impaired phagocytic clearance of apoptotic cells and subsequent accumulation of inflammatory cellular contents, including lipids and lysosomal enzymes [21–23].

Studies of circulating monocytes are important, yet, these cells are short-lived and are distinguishable from their progeny macrophages in plaques. Purification of viable, fully functional macrophages from plaques is difficult; therefore, we sought to characterize the phenotype and function of monocyte-derived macrophages (MDMs) from ART-treated PWH and from individuals without HIV to study the function and transcriptional profiles of these cells in more detail. We hypothesized that pro-atherogenic MDM phenotypic and functional profiles would be related to circulating biomarkers associated with enhanced CVD risk in PWH. We identified complex transcriptional, phenotypic and functional differences between MDMs from people with and without HIV, including an increased propensity of MDMs from PWH to form foam cells and produce mediators of vascular inflammation (i.e. IL-1β, TNFα, ROS, and MMPs). MDM activation profiles were likely driven by serum factors, including perturbations in the lipidome in PWH. Greater understanding of the pro-atherosclerotic transcriptional and functional phenotypes of MDMs in PWH may provide targets for reducing ASCVD in PWH and in the general population.

## Results

Demographic information for study participants with (N = 25) and without (N = 20) HIV is provided in **Table 1**. Prior to enrollment, all participants provided written informed consent. All PWH were on suppressive ART (HIV-1 RNA <40 copies/mL) and had a mean CD4+ T cell count of 627 cells/μL. The levels of total cholesterol (TC), LDL, and triglycerides (TG) were not statistically different between study populations; PWH had reduced levels of high-density lipoprotein (HDL, p = 0.001) compared to levels among individuals without HIV. PWH had an average ASCVD 10-year risk score of 6%.

**Table 1:** Study Participant Demographics. Demographics and clinical characteristics of study participants with and without HIV. Data are displayed as means and ranges; age values also include standard deviation. (N/A, Not Available).

	PWH (n = 25)	HIV- (n = 20)
Age	44+/-15 (20–62)	36+/-12 (21–68)
CD4+ T Cell Count (cells/uL)	627 (296–1025)	N/A
HIV Viral Load (copies/mL)	<40	N/A
On ART (%)	100%	N/A
Sex (%)	Male = 88% Female = 12%	Male = 85% Female = 15%
Ethnicity (%)	White = 68% Non-White = 32%	White = 75% Non-White = 25%
Total Cholesterol (mg/dL)	169 (100–256)	177 (130–254)
HDL (mg/dL)	44 (23–58)	52 (32–76)
LDL (mg/dL)	92 (34–159)	93 (25–162)
Triglycerides (mg/dL)	226 (51–737)	151 (45–348)
Current Smokers (%)	28	15
Statin Use (%)	36	13
ASCVD 10-year CVD risk score (%)	6 (3–12)	N/A

### MDMs from PWH have altered transcriptomes and cell surface phenotype

**Fig 1:**
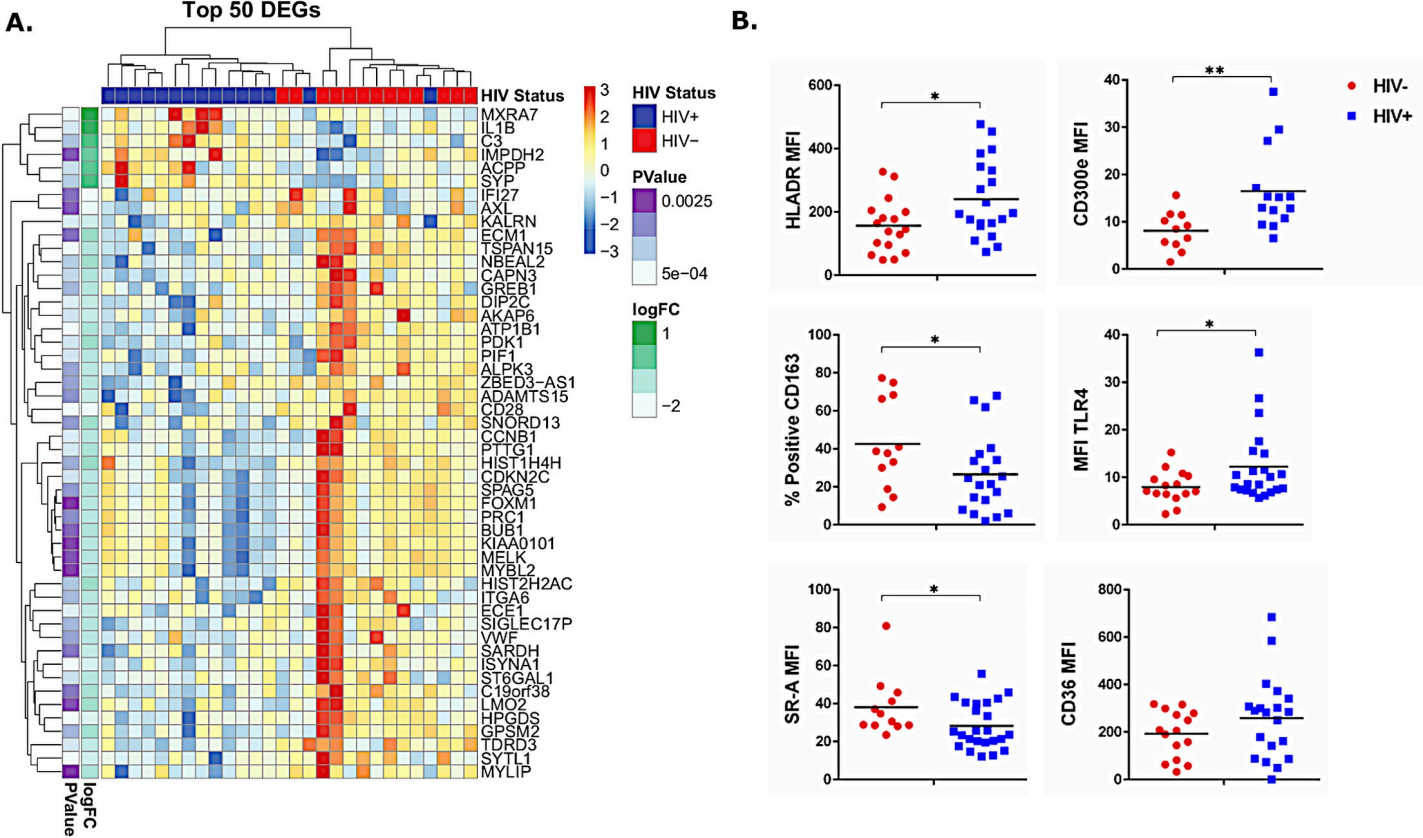
Altered macrophage phenotype in HIV+ individuals may contribute to vascular inflammation. A) Transcript analysis identified differentially expressed genes among macrophages obtained from donors with and without HIV. Data are arranged by p-value and log fold change (LogFC). B) Surface activation marker expression measured by flow cytometry. *p<0.05, **p<0.01.

MDMs from 15 PWH and 13 controls revealed distinct transcriptional profiles; the demographics of this subset were similar to the overall group. We identified 811 differentially expressed genes (DEGs) (p<0.05) and 79 DEGs with an adjusted p value <0.05. The top 50 DEGs are visualized in **Fig 1A**. Notably, expression of matrix remodeling gene, MXRA7, ACPP, a gene upregulated in response to lipid accumulation[24], and IL1β, were increased in MDMs from PWH. We measured decreased expression of cell cycle regulatory genes CDC20, CDKN3, and CCNB1 in MDMs from PWH relative to levels in cells from individuals without HIV. We also observed multiple innate immune signaling and lipid sensing pathway alterations between groups, including IL-10, wnt, glucocorticoid receptor signaling, and LXR/RXR signaling. These gene expression profiles are suggestive of a pro-inflammatory, pro-atherosclerotic phenotype of MDMs in PWH [16].

Monocyte subset proportions are altered in PWH and have differential expression of activation and adhesion molecules [13, 25–27]. Here, we confirm enrichment of inflammatory (CD14^+^CD16^+^) and patrolling (CD14^dim^CD16^+^) monocytes, and increased levels of innate immune receptors and activation markers, including TLR4, SR-A, CD163, Tissue Factor (TF), and CD36 on monocytes from PWH compared to those from people without HIV. Levels of the activation marker, CD300e, are also increased on inflammatory (CD14+CD16+) and patrolling (CD14^dim^CD16^+^) monocyte subsets in ART-treated PWH (**S1A Fig**).

We hypothesized that MDMs from PWH may have similar alterations in cell surface phenotype. After 5 days of differentiation in autologous serum, we measured increased levels of HLA-DR (p = 0.02), TLR4 (p = 0.05), and CD300e (p = 0.008), and decreased levels of CD163 (p = 0.05) (**Fig 1B**). Although we measured increased levels of adhesion molecules on monocytes directly ex vivo (**S1A Fig**), expression of CD11a, CD11b, and CD11c did not differ between MDMs from individuals with or without HIV. We did not detect differences in levels of CD69, TF, and CD86 on MDMs from individuals with or without HIV (**S1B Fig**).

### Altered macrophage function in PWH may contribute to vascular inflammation

**Fig 2:**
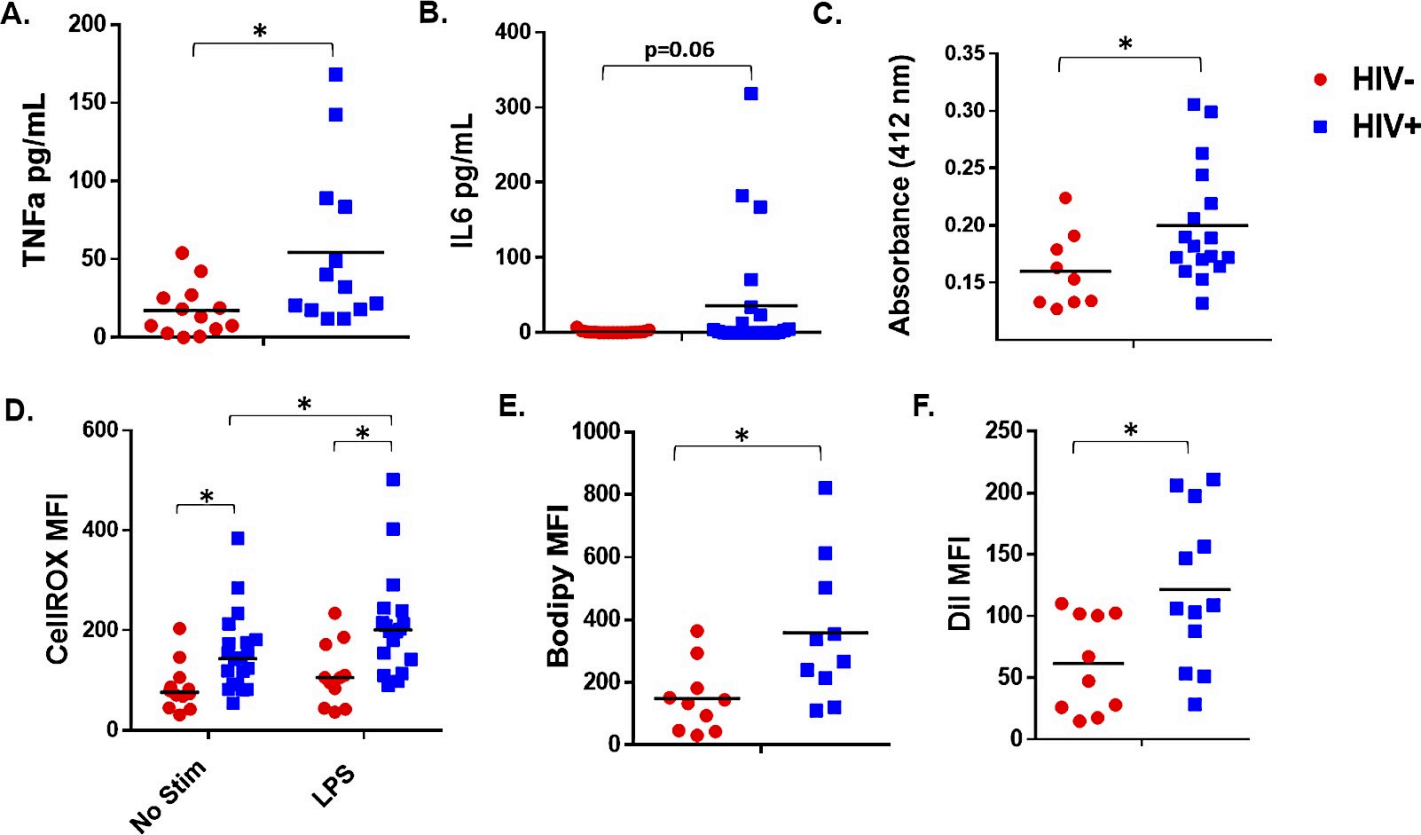
Altered macrophage function in PWH may contribute to vascular inflammation. Inflammatory cytokine production, A) TNFα and B) IL-6, was measured in MDM supernatants (24 h) by ELISA. C) Total MMP activity was measured in supernatants (24 h) from HIV- and HIV+ MDMs. Increased absorbance indicates greater MMP activity. D) MDMs were cultured in medium containing autologous serum alone (20%), or stimulated with LPS for 1.5 h (100 ng/mL). Cells from HIV- and HIV+ donors were incubated with CellROX Deep Red, and intracellular production of ROS was measured by flow cytometry. E) Intracellular lipid accumulation was measured using Bodipy (493/503) stain and flow cytometry. F) Lipid uptake was measured by flow cytometry following exposure (4 h) of MDMs to labeled DiI-OxLDL. (p<0.05).

We next assessed functional differences in MDMs obtained from individuals with and without HIV. MDMs from PWH spontaneously produced more pro-inflammatory cytokines, including TNFα (p = 0.03) and IL-6 (p = 0.06), than MDMs from individuals without HIV (**Fig 2A and 2B**). We also detected increased MMP activity in supernatants collected from MDMs from PWH compared to activity levels produced by MDMs from individuals without HIV (**Fig 2C**). MMPs are proteolytic enzymes important in vascular remodeling and rupture of atherosclerotic plaques, and may serve as important biomarkers to predict CVD events [28, 29]. MDMs from PWH also produced more ROS spontaneously (p = 0.01), and in response to LPS (p = 0.007) (**Fig 2D**). Monoclonal antibody-mediated activation of CD300e elicited ROS production by MDMs from individuals without HIV, suggesting that CD300e pathway activation may warrant further investigation in development of ASCVD in PWH, as ROS production may contribute to lipid oxidation and vascular inflammation, and CD300e is increased on MDMs from PWH (**S2A Fig**).

We next measured intracellular lipid accumulation and foam cell formation using the fluorescent lipophilic dye, Bodipy. MDMs from PWH displayed increased Bodipy uptake compared to MDMs from subjects without HIV (**Fig 2E**). Further, we exposed MDMs to DiI-labeled oxLDL and measured oxLDL uptake [30]. After 4 h exposure, DiI-oxLDL levels were greater in MDMs from PWH, indicating enhanced foam cell formation (**Fig 2F**). In addition to lipid uptake, macrophage phagocytic capacity is reportedly altered in advanced atherosclerotic plaques [31]. We did not detect differences in phagocytosis of *E*. *coli* bioparticles between MDMs from individuals with and without HIV (**S2B Fig**).

### Inflammatory biomarkers associated with morbidity and mortality in HIV infection correlate with unique DGE signatures and altered pathway activation

**Fig 3:**
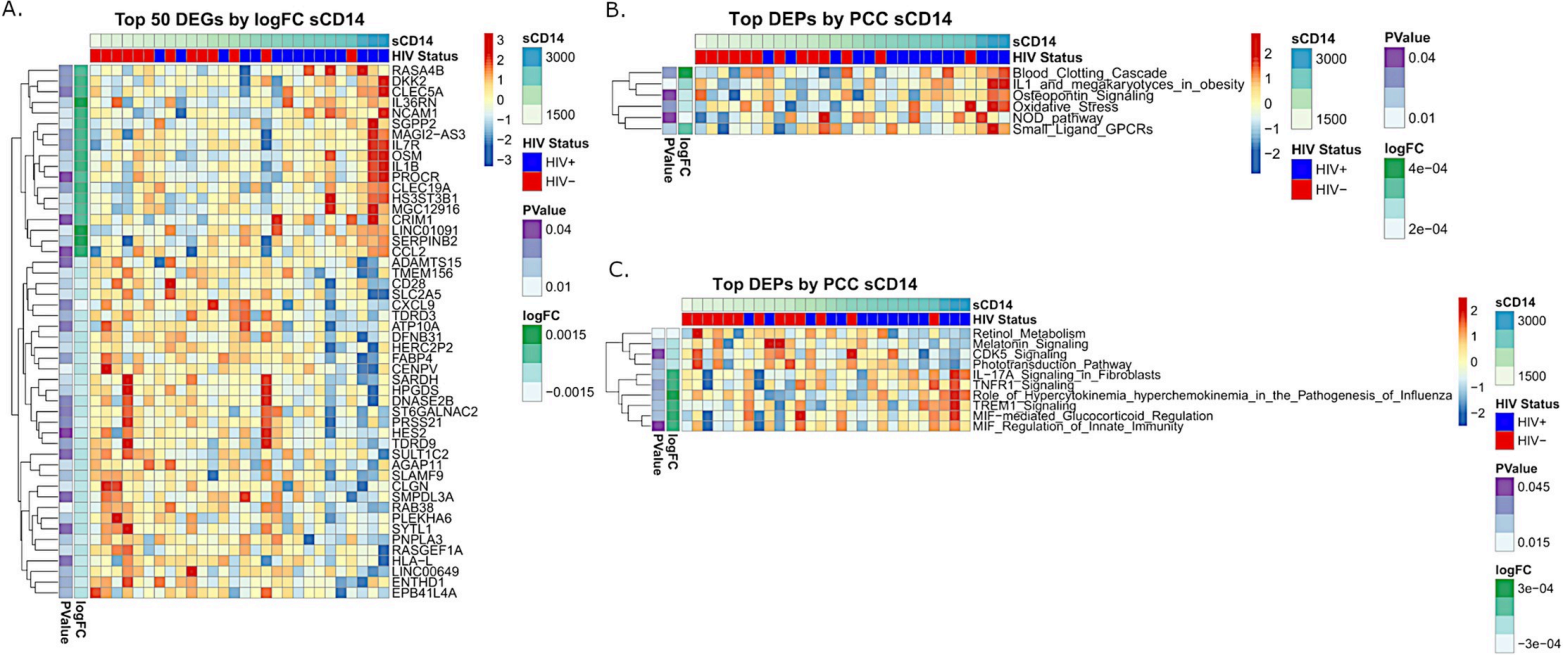
Biomarkers associated with morbidity and mortality in HIV infection correlate with unique DGE signatures and altered pathway activation. A) Top 50 differentially expressed genes regressed against donor sCD14 serum levels. B) Pathways identified using gene set variation analysis (GSVA) using the regressed genes selected above from donor sCD14 serum levels and ranked by P value (P≤0.05). C) Differentially expressed pathways (DEPs) were identified using GSVA and ranked by PCC (P≤0.05).

Macrophages are heterogenous cells that continually adapt their phenotype and function to progressive changes in microenvironment [14–17]. We differentiated MDMs in culture medium containing autologous serum, as opposed to a standardized medium containing growth factors and exogenous cytokines that artificially skew macrophage phenotype [32, 33], and hypothesized that differential activation of MDMs from PWH is likely due to drivers of immune activation present in donor serum. As expected, we measured increased levels of markers of inflammation (TNFR1, TNFR2, IL-6), endothelial activation (ICAM-1, VCAM-1, CX3CL1), monocyte/macrophage activation (sCD14, sCD163), microbial translocation (LBP), oxidative stress (MPO), and coagulation (D-dimer) in serum from PWH compared to levels in serum from individuals without HIV (**S3 Fig**). Serum levels of inflammatory biomarkers associated with morbidity and mortality in HIV infection, including sCD14, sCD163, and TNFR2 [10, 11], directly correlated with unique MDM DGE signatures, including MMP expression, inflammatory signaling, oxidative stress pathways, and coagulation activation (**Fig 3A–3C, S4A–S4C Fig**). Furthermore, transcript levels of SREBP1, SPON2, and MT1, genes associated with lipid accumulation in macrophages [24, 34], were also altered in PWH and correlated with inflammatory biomarkers (sCD14, **Fig 3A–3C;** sCD163, TNFR2, and ROS, **S4A–S4C Fig,** respectively).

### Increased levels of ceramides are associated with pro-atherosclerotic MDM transcriptional profiles

Certain lipid classes and lipid species are associated with CVD in PWH and in the general population, and may drive vascular inflammation and myeloid cell activation [27, 35–39]. Elevated levels of ceramides (CER) are associated with ART use and carotid atherosclerosis in PWH [40], and with CVD events in individuals without HIV [41]. We measured increased levels of CERs in PWH, and when we stratified MDM transcriptional profiles based on CER levels, increasing concentrations of CERs were associated with activation of the intrinsic and extrinsic coagulation pathways, increased G-protein coupled receptor signaling, and decreased fatty acid metabolism, nicotinamide metabolism, and eicosanoid signaling in MDMs (**S5A Fig**). These gene expression and pathway analyses provide mechanistic insight into CVD progression associated with alterations in lipid species and lipid classes.

Enrichment of CD16^+^ vascular homing monocytes in circulation may contribute to macrophage populations in the vessel wall with differential functions. In this study, increased CD16^+^ monocyte subset proportions in PWH directly correlated with altered inflammatory signaling and reduced cell cycle regulatory mechanisms in MDMs derived from these donors (**S6A Fig**). Altered proportions of circulating monocytes may have important implications for the subsequent differentiation and polarization of tissue resident macrophages. Further studies on purified cell populations are warranted.

### Exposure of MDMs from HIV-negative participants to pooled serum from PWH is sufficient to alter macrophage phenotype

**Fig 4:**
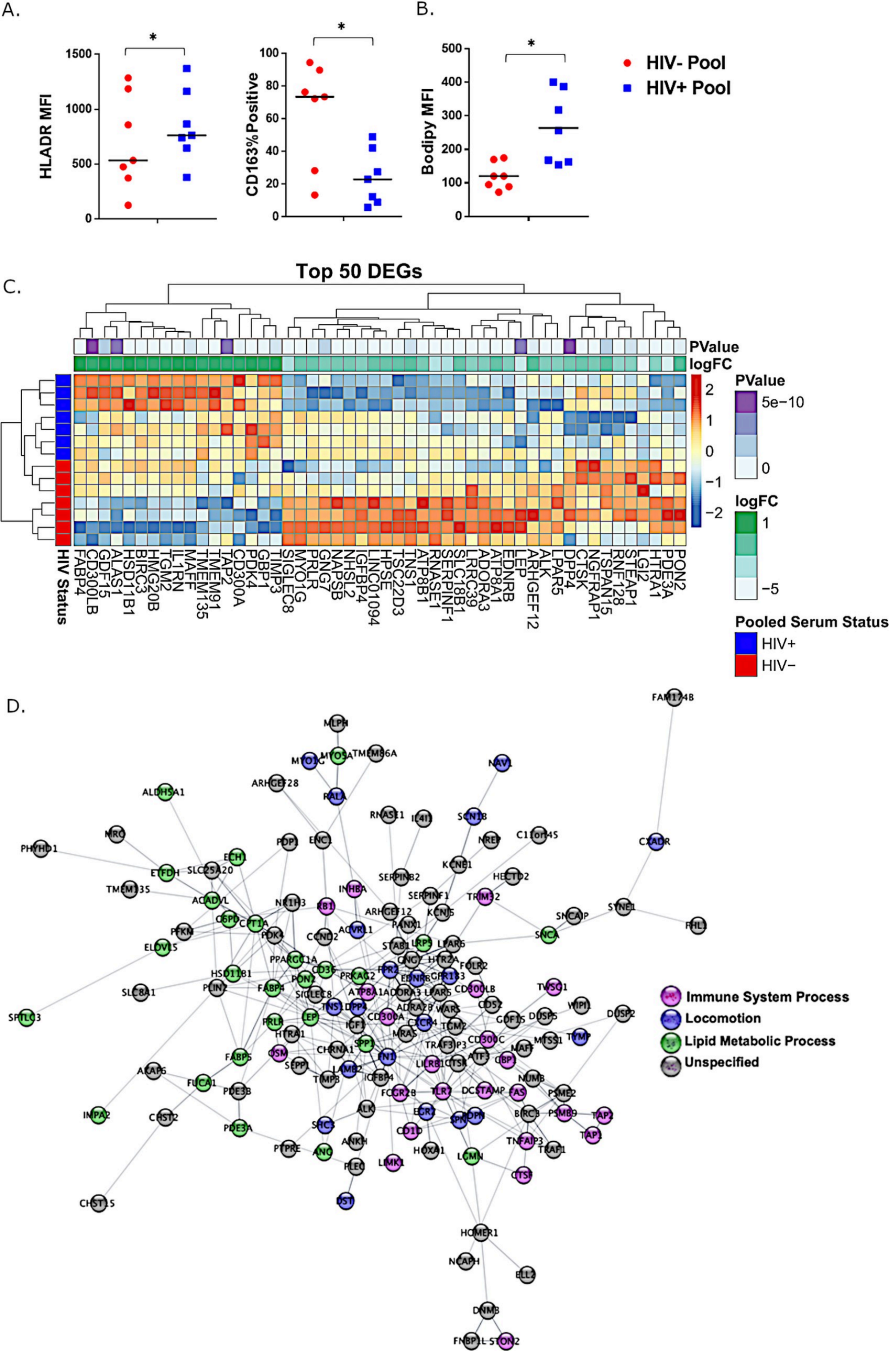
Exposure to pooled serum from PWH is sufficient to alter macrophage phenotype. A) PBMCs from HIV- donors were cultured for 5 days in either HIV- or HIV+ pooled serum to obtain MDMs. Surface expression of activation markers, HLADR and CD163, were measured by flow cytometry. B) Intracellular lipid accumulation was assessed by flow cytometry following staining with Bodipy (493/503). C) Differential gene expression was analyzed from RNA isolated from MDMs differentiated in HIV- or HIV+ pooled serum. D) Integrative analysis for HIV+/- pooled serum induced transcriptional changes. Network DGE; p<0.005.

To determine whether serum factors are sufficient to drive alterations in MDM phenotype, we obtained cells from individuals without HIV and differentiated these cells in the presence of serum pooled from either HIV-uninfected donors (N = 7) or HIV+ ART+ donors (N = 7). Consistent with the phenotype of MDMs from PWH, we measured significantly increased levels of HLA-DR and reduced CD163 on MDMs grown in pooled serum from HIV+ donors compared to those grown in serum from HIV- donors (**Fig 4A**). Furthermore, we detected increased intracellular lipid accumulation in MDMs grown in HIV+ pooled serum compared to lipid accumulation by cells grown in HIV- pooled serum (**Fig 4B**). Transcriptomic analyses of MDMs grown in HIV- or HIV+ pooled serum revealed significant differences in gene expression (2675 DEGs, p<0.05; 1192 DEGs adjusted p<0.05). The top 50 significant DEGs are represented as a heatmap in **Fig 4C,** including increased expression of FABP4 and GDF15, biomarkers of CVD [42, 43]. We also identified altered expression of genes involved in fatty acid metabolism pathways, interferon signaling, and MAPK signaling. Among the genes that were upregulated (N = 16) and downregulated (N = 64) in both MDMs from PWH and in HIV- cells exposed to HIV+ pooled serum were genes associated with innate signaling/inflammation (TNFAIP8L2, TNFSF14, SMAD6, IL1RN, INHBA) and metabolism/lipid processing (LDHD, LEP, RETN, GBE1).

Pooled serum from HIV+ ART+ donors was enriched for SaFAs, containing significantly elevated concentrations of myristic acid (14:0), palmitic acid (16:0), margaric acid (17:0), and stearic acid (18:0) compared to levels of these lipids in HIV- pooled serum (**S7A and S7B Fig**). SaFAs promote inflammation [39], and are associated with immune activation in HIV infection [36]. Pooled serum from HIV+ donors also had decreased levels of anti-inflammatory PUFAs [44, 45]. Surface expression of the activation marker HLA-DR on macrophages grown in HIV+ pooled serum was directly associated with levels of free SaFAs and inversely related to free PUFA levels (**S7C Fig**).

In order to gain a greater appreciation of the broad gene expression changes induced by differentiating MDMs in pooled serum from PWH, we performed network analyses (**Fig 4D**). Differential gene expression clustered around several main “hubs”; this clustering identified changes in gene expression associated with innate immune activation (TLR7, CD300s, INHBA, etc), coagulation (SERPINB2, SERPinF1), and lipid and scavenger receptors (CD36, MARCO). This network analysis also shows activation of multiple signaling cascades and regulators of gene expression, including expression of genes related to peroxisome proliferator-activated receptor beta/delta (PPARβδ). Overall, this network of gene expression changes induced by pooled serum from PWH demonstrates alterations in multiple pathways that likely contribute to a highly activated MDM phenotype with dysregulated lipid processing. Many of these pathways are differentially activated in MDMs from PWH, suggesting that serum factors in PWH may induce global gene network changes, skewing these cells toward a pro-coagulant, pro-atherosclerotic phenotype.

### Systems model of MDM phenotype in PWH

**Fig 5:**
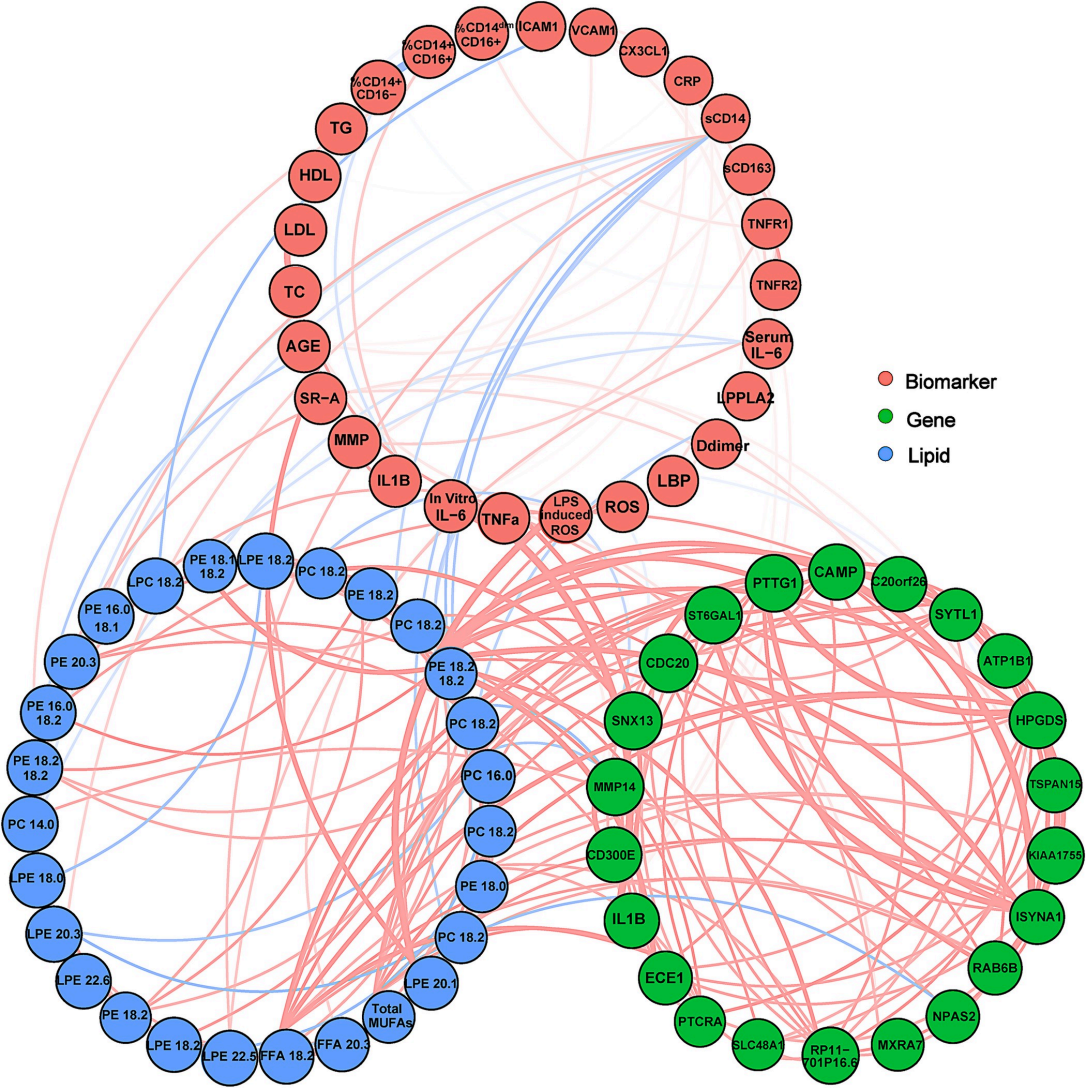
Transcriptomic, lipidomic and protein expression model. We performed linear regression to identify genes, lipids and proteins that are most highly predictive for all three data types. The most highly interactive genes and lipids are displayed as nodes, with edges representing Pearson correlations between genes and lipids, and genes and biomarkers, as well as PCCs for actual gene-gene correlations. Because the lipid and gene data was so highly interactive, only edges with p-value < 0.01 and have an r-value > 0.5 were shown for clarity.

To integrate our transcriptomic, lipidomic, and protein expression data into a full systems level model, we performed linear regression to identify genes, lipids and proteins that are most highly predictive for all three data types. In **Fig 5**, we display the most highly interactive genes and lipids as nodes, with edges representing Pearson correlation coefficients (PCCs) between genes and lipids, and genes and biomarkers, as well as PCCs for actual gene-gene correlations. This integrative analysis allowed us to identify additional genes that are not simply differentially regulated in macrophages from PWH, but also are most predictive of differential lipid levels, and may play roles in differential PPAR signaling, macrophage function and differentiation, and as such, are potential therapeutic targets to be validated and explored in our future studies. For example, Hematopoietic prostaglandin D synthase (HPGDS) expression is decreased in MDMs from PWH, and is known to be modulated via PPAR gamma and LPS, and plays a pivotal role in macrophage migration [46]. Solute Carrier Family 48 Member 1 (SLC48A1), also known as Heme Transporter HRG1, is a differentially regulated gene in plaque rupture, and is also strongly correlated in our model with lipid levels in PWH [47]. Our systems level model identified additional genes with epigenetic regulatory functions that were significantly correlated with lipidome measurements, such as Disco Interacting Protein 2 Homolog C (DIP2C) [48] and TDRD3 [49–51]. These genes may contribute to the macrophage response to circulating lipid levels.

We have also highlighted a number of other selected genes that may drive changes within atherosclerotic plaques in PWH (see Highlighted Genes, **Fig 5**). For example, the gene for ST6 β-galactoside α-2,6-sialyltransferase1 (ST6GAL1) is associated with increased fat droplet formation, inflammatory cytokine expression and promotion of a pro-inflammatory M1 macrophage phenotype in mouse models [52]. Pituitary Tumor-Transforming gene 1 (PTTG1) was also significantly correlated with lipid levels in PWH and regulates myeloid cell differentiation [53]. MMP14, which contributes to plaque rupture and angiogenesis, was highly correlated to overall concentrations of PE lipids, and FFA(16:0) and FFA(22:2) in our model, as well as CD300E gene expression [54]. We also identified Matrix remodeling associated 7 (MXRA7) in our integrative analysis, a protein that has been shown to regulate the expression of other matrix remodeling-related genes (fibronectin and TIMP1) and may form an important link between lipid metabolism and matrix remodeling within the plaque [55]. We plan to validate these highly significant associations further, and propose that this level of systems analysis will greatly aid efforts to identify novel mechanisms driving pro-atherogenic MDM phenotypes in PWH.

## Discussion

ASCVD risk is increased in PWH, even during suppression of HIV replication with ART [3, 56, 57]. Activation of the endothelium and infiltration of immune cells are important contributors to the development of atherosclerosis [8]. Previous studies have demonstrated differential expression of chemokine receptors and adhesion molecules on monocytes in PWH [10, 13, 25, 26, 58, 59], suggesting that altered monocyte activation and increased vascular homing of these cells may contribute to atherosclerosis in this population. Following migration of circulating monocytes into the vessel wall, these cells differentiate into macrophages that can exacerbate atherosclerotic lesion development through mechanisms associated with dysregulated lipid accumulation and production of several pro-inflammatory mediators [18, 19]. Here, we investigated phenotypic, transcriptional, and functional abnormalities in MDMs from ART-treated PWH that may contribute to increased incidence of CVD in this population.

Plaque macrophages exhibit a dynamic phenotypic range, driven by stimuli in the microenvironment, with complex signaling mechanisms mediating macrophage plasticity and polarization [60]. Cells purified from atherosclerotic plaques may be more proximal to CVD progression, yet, acquisition of MDMs within atherosclerotic plaques is difficult. These cells may lose functional capabilities and may not retain their phenotypes during isolation. To better study macrophage phenotype in ART-treated people with HIV, we used a model system in which MDMs are differentiated in the presence of autologous serum for 5 days [32, 33], enabling us to explore the contributions of multiple serum components (i.e. the lipidome, inflammatory cytokines, microbial products) on MDM profiles. This method reduces artificial cellular activation induced by non-physiological concentrations of growth factors that skew these cells along artificial differentiation pathways, masking relevant gene and protein expression differences in cells from subjects with and without HIV. Analysis of sera isolated from PWH revealed increased levels of biomarkers of immune activation and altered lipidome composition compared to levels of these indices in sera from individuals without HIV. Exposure to components found in the chronic inflammatory environment in HIV infection [4, 7, 61] likely plays a role in promoting atherogenic macrophage phenotypes.

Macrophages in rupture-prone plaque regions were previously found to have a predominantly pro-inflammatory phenotype, whereas anti-inflammatory phenotypes were associated with plaque stability [62]. Here, we demonstrated that MDMs from PWH express a transcriptional profile that is associated with increased innate immune receptor activation and production of pro-inflammatory mediators including MMPs and IL-1β. We also determined that MDMs from PWH spontaneously, and in response to LPS, produce more ROS than do cells from individuals without HIV. Our data suggest that increased CD300e expression on MDMs from PWH may also contribute to increased ROS and oxidative stress in PWH, a novel pathway that needs to be more fully explored. Historically, macrophages have been categorized into two phenotypes, the classically activated macrophage (M1) and the alternatively activated macrophage (M2) [63]. The MDM phenotype we observe from PWH more closely resembles a pro-inflammatory M1 macrophage; however, aspects of M2 macrophages are also present, and classifications of macrophages into M1 and M2 phenotypes do not fully describe the diversity of macrophage populations in vivo [33, 64]. Our comprehensive systems level analyses better describes the spectrum of macrophage activation in PWH.

Oxidative stress in vessel walls promotes inflammatory modification of LDL which is recognized by pattern recognition receptors (PRRs) on innate immune cells [65]. Scavenger receptors, including scavenger receptor-A1 (SR-A) and CD36, are PRRs on macrophages involved in modified lipid uptake and foam cell formation [66]. *In vitro*, SR-A and CD36 mediate 75–90% of the internalization of modified LDL [67]. We measured increased lipid accumulation and more rapid uptake of DiI-labeled oxLDL in MDMs from the PWH, indicating an increased propensity for foam cell formation. These data are consistent with a previous study [68]. Mechanistic insights into the increased lipid accumulation within MDMs from PWH are also generated through identification of changes in transcript levels of genes associated with lipid uptake and processing, including expression of SREB1, MT1, and ACPP, as well as changes in RXR/LXR signaling.

We also measured increased expression of SR-A and CD36 on monocyte subsets from PWH directly ex vivo, however, after differentiation of MDMs, we did not detect significant differences in CD36 levels, and surface expression of SR-A was reduced on MDMs from PWH. Previous studies have measured both decreased [68, 69] and increased [70, 71] scavenger receptor expression in response to oxLDL uptake, and the kinetics of scavenger receptor expression during foam cell formation are incompletely understood. Future studies should measure surface levels of additional scavenger receptors, including SR-B, MARCO, LOX1, and SREC-1 [66], and explore mechanisms of foam cell formation in MDMs from PWH. Importantly, SR-A is involved in a broad range of immune functions, including recognition of apoptotic cells and inflammatory signaling, and reduced SR-A levels may result in significant consequences for macrophage activation. The protein, gene, and signaling cascade data presented here demonstrate that alterations in innate immune receptor signaling are likely important in the pro-atherosclerotic phenotype of MDMs in PWH.

The underlying drivers of increased innate immune activation in MDMs from PWH are myriad and include microbial translocation, coinfections, and inflammatory lipid profiles [72]. Previous studies have linked lipid species and lipid classes to markers of immune activation in PWH [36, 38, 72]. Here, this work extends those findings and provides multidimensional associations among the lipidome, inflammatory markers, and MDM transcriptional and functional profiles. Importantly, levels of SaFAs and CERs were directly related to markers of inflammation (TNFR1 and 2, IL-1β), monocyte activation (sCD163, sCD14, % of inflammatory monocytes), and pro-atherogenic MDM phenotype and function (MMP production and lipid processing). Conversely, levels of PUFAs, such as FFA(18:3) and LPC(18:2), are inversely associated with inflammation and MDM activation. These data provide insights into how alterations in the lipidome and inflammation, induced by HIV and ART, could contribute to CVD progression through activation of innate signaling pathways in MDMs.

These associations are bolstered by our *in vitro* experiments in which monocytes from donors without HIV were differentiated in pooled serum samples from PWH. The network of interactions among the most highly differentially regulated genes in response to HIV+ serum exposure illustrates an interplay between lipid signaling and soluble serum factors in PWH (**Fig 4**). Exposure to serum from PWH strongly induced the transcriptional signature of peroxisome proliferator-activated receptor beta/delta (PPARβδ) axis activation, known to regulate macrophage polarization and metabolism in response to lipid ligands [73]. PPARβδ activity robustly targets genes involved in fatty acid metabolism and lipid accumulation, such as ACADVL, CPT1A, ECH1, GDF15, PDK4, CPT1a, SLC25A20, FABP4, CD36, and PLIN2, all of which were upregulated in response to serum from PWH [74]. PPARβδ target genes also have diverse immunomodulatory roles, and levels of many of these genes were also significantly altered in MDMs exposed to serum from PWH on ART, including CD300A, CD300LB, CD32B, CD52, and MAP3K8 [73]. Also altered in response to serum from PWH was LRP5, a member of the LDL receptor superfamily associated with inflammatory macrophages in advanced atherosclerotic lesions [75], ST14, a mediator of extracellular matrix degradation and infiltration of plaque macrophages in atherosclerosis [76], and CD1D, which codes for an MHC-related surface receptor that binds and presents lipid antigens and participates in PPARγ activation via oxidized cholesterol [77]. Additionally, altered regulation of the inflammasome signaling cascade was evident in MDMs exposed to serum from PWH. We measured increased levels of NLRC4, which indirectly senses pathogen-associated molecular patterns (PAMPs) and assists in the assembly of the inflammasome complex to initiate caspase-1-mediated pyroptosis [78]. PPARβδ-mediated networks may be important mediators of macrophage phenotype and function in PWH.

Several plasma biomarkers have been associated with all-cause mortality (IL-6, D-dimer, hsCRP, TNFR1 and 2) and CVD risk and progression (sCD14, sCD163) in PWH [5, 6, 61, 79, 80]. Currently, the association between any of these markers and morbidity/mortality is speculative, with little mechanistic clarification. Here, we demonstrate that biomarkers associated with morbidity and mortality in PWH, and in HIV- populations, are associated with MDM functional and transcriptional profiles associated with lipid processing, coagulation, inflammation, and ROS and MMP expression. The associations between individual biomarkers and specific gene and signaling cascades were identified in an unbiased manner. Further exploration of these pathways and development of targeted intervention strategies could enhance dramatically our ability to reduce CVD risk and improve the health of both PWH and aging populations without HIV.

Given the critical role of MDMs in atherosclerosis, these cells are potential targets for therapeutic intervention. Modulation of inflammatory signals may play a key role in therapeutic strategies to mitigate atherosclerosis [81]. The Canakinumab Antiinflammatory Thrombosis Outcome Study (CANTOS) trial demonstrated that inhibiting IL-1β pathway activation significantly reduced coronary artery disease morbidity and mortality in HIV uninfected persons [82]. Additionally, statin use improves lipid profiles and reduces inflammatory markers and CVD risk in PWH [83]. Our work identifies potential targets for altering MDM function to reduce CVD progression in PWH, including inhibition of innate immune receptors (CD36, TLRs, SRA, CD300), improving the altered lipid profile (decreasing SaFAs and CERs, and increasing PUFAs) or through modulation of signaling cascades associated with inflammation and metabolism (NFκB, the coagulation cascade, and PPARβδ). An improved understanding of the drivers of macrophage activation may lead to better strategies for treatment of chronic inflammation and CVD in PWH.

Using this autologous serum based culture system to study macrophages from PWH receiving ART, our integrative systems model has identified profile of MDM phenotype, function, gene expression, and signaling cascade activation that may drive increased CVD incidence in PWH. While several of our analyses are performed independent of HIV serostatus, it is worth noting that our two groups may not be ideally matched on traditional CVD risk factors, including diet, smoking status, statin use, exercise, and coinfections with other persistent viral infections including cytomegalovirus. Thus, we cannot exclude the possibility that the differences we observed are due to other confounding factors besides HIV. While age ranges were similar between groups, our PWH tended to be older. Interestingly, among PWH the proportion of CD14+CD16+ monocytes tended to be inversely related to age (r = -0.397, p = 0.08), suggesting that other important factors likely drive monocyte/MDM activation in this population. We also explored associations among age and our transcriptomic data; while some genes were associated with age (including CDC20 CDKN3, SPON2, PTCRA), many other genes were not (IL-1β MXRA7, ACCP, CD300, MMP14, INHBA, LDHD, ST6GAL1, PTTG; S1 Table). Statin use was also more prevalent among our PWH, yet these study participants displayed increased inflammatory biomarkers and altered lipidome composition. Further study is necessary to build and better define integrative models of macrophage activation in CVD progression in both HIV infection and in HIV- populations, including more direct measurement of CVD progression by coronary calcium scoring or coronary plaque volume, and to better understand the complex mechanisms that drive macrophage activation *in vivo*. This work moves our understanding of this field forward, and identifies multiple directions for future study and potential intervention.

## Materials and methods

### Study participants

All participants provided written informed consent in compliance with the Ohio State University Institutional Review Board (2014H0467). Participants were recruited by convenience, with loose matching on demographics throughout enrollment. People with HIV were recruited from the OSU Infectious Disease Clinic, people without HIV were typically recruited from among staff members at OSU Wexner Medical Center. Demographic information, HIV-1 RNA, CD4+ T cell counts, TC, HDL, LDL, and TG levels were extracted from medical charts for each participant with HIV from the most recent time point available. ASCVD risk score was calculated for PWH [84].

### Isolation and culturing of human monocyte-derived macrophages

Blood was collected in EDTA-vacutainer tubes (BD Biosciences) for peripheral blood mononuclear cell (PBMC) separation. Serum was collected from blood using 10 mL serum separating tubes (SST, BD Biosciences) and centrifuged for 15 min at 800 x g. PBMCs were isolated from donors without HIV (N = 20) and ART-treated PWH (N = 25). For differentiation of monocyte-derived macrophages (MDMs), PBMCs (2 x 10^6^/mL) were cultured in Teflon wells (Savillex) for 5 days in RPMI 1640 supplemented with 20% autologous serum. Teflon wells were chosen for differentiation of macrophages because cells cultured in plastic dishes may display artificially increased activation profiles compared to cells cultured in Teflon [85, 86]. On day 5, cells were removed from Teflon and MDMs (3 x 10^5^/mL) were purified by adherence to plastic for 3 h. Supernatants were collected after 24 h to measure TNFα and IL6 production. Among study participants with and without HIV, MDM yield on day 5 was approximately 10% of total PBMCs.

For MDM pooled serum experiments, PBMCs were isolated from donors without HIV (N = 7) and differentiated in Teflon wells for 5 days in 20% serum pooled from donors with (N = 8) or without (N = 8) HIV. For CD300e activation experiments, tissue culture plates were coated with 1 μg/mL of anti-CD300e monoclonal antibody (clone UP-H2, Abcam) [87] or isotype-matched control (IgG1) and incubated for 2 h at 37C. MDMs were added to the coated wells and cultured for 24 h. Differentiation of monocytes into macrophages was confirmed by flow cytometry and by microscopy.

### Flow cytometry

PBMCs harvested from Teflon were washed and blocked in 10% human AB serum (Sigma) for 60 min. Cells were then stained for 30 min in the dark on ice, washed, and fixed in 1% paraformaldehyde. Monocytes and MDMs were identified by granularity, size, and surface expression of CD14 and CD16 (anti-CD14 pacific blue and anti-CD16 PE, BD Pharmingen). MDM surface markers were measured using fluorochrome-labeled antibodies: HLA-DR (APC-Cy7), CD69 (PE-Cy7), CD18 (FITC), CD11a (PE-Cy7), CD11b (PE-Cy5), CD11c (APC), CD36 (APC), TLR4 (PE-Cy7) (BD Pharmingen); CD163 (PE-Cy5) (eBioscience); Tissue factor (FITC); SR-A (FITC), CD300e (PE-Cy7) (Miltenyi).

### RNA isolation and gene expression analysis

Total MDM RNA was extracted using RNeasy spin kits (Qiagen). RNA quality evaluation and quantification were performed on Advanced Analytical’s Fragment Analyzer using the Standard Sense RNA assay. RNA-Seq libraries were prepared with Illumina's TruSeq Stranded Total RNA (Ribo-Zero Globin) kits. Libraries were sized and assessed on Advanced Analytical's Fragment Analyzer with the High Sense NGS assay and quantified using a Life Technologies' Qubit 3.0 flourometer. Sequencing was performed with an Illumina HiSeq 2500 instrument on a High Output flow cell (2 x 50 cycle, 30M+ paired reads/sample). Raw demultiplexed fastq paired end read files were trimmed of adapters and filtered using the program skewer to remove reads with an average phred quality score of less than 30 or trimmed to a length of less than 36 [88]. Trimmed reads were then aligned using the HISAT2 aligner to the Homo sapiens NCBI reference genome assembly version GRCh38 and sorted using SAMtools [89, 90]. Aligned reads were counted and assigned to gene meta-features using the program featureCounts as part of the Subread package [91]. These files were imported into the R programming language and were assessed for quality, normalized, and analyzed for differential gene expression testing using the edgeR Bioconductor library [92]. Gene set variation analysis was also performed on the data using the GSVA Bioconductor library and the Molecular Signatures Database v5.0 [93, 94]. Linear regression modelling was performed using the limma framework [95]. Protein-protein interaction visualizations were generated using Stringdb and perfuse-force class directed functional enrichment of differential gene expression was performed using GOlorize and BINGO to determine Gene Ontology and map functional groups [96, 97]. The integrative model was created by first calculating the average Pearson correlation coefficient (PCC) of each lipid to the top differentially expressed genes between PWH and HIV- groups at a p value < 0.01. The absolute average correlation was then used to rank the lipids and the top 50 were included in the model. Individual PCCs were then calculated between the top 50 lipids, biomarkers, DEGs previously mentioned, as well as the following genes that were selected from the differential gene expression and/or protein analyses: PPARD, IL1B, CD300E, MMP14, SREBF1, SPON2, SNX13, and CDC20. Nodes and edges indicating PCC were plotted using the qgraph package [98]. All heatmap figures show genes and pathways that pass a nominal p < 0.05 unless stated otherwise. Complete RNAseq data archived under Gene Expression Omnibus (GEO) accession ID#: GSE157884.

### Soluble markers

Levels of TNFα and IL6, were measured in MDM supernatants by ELISA (R&D Systems unless stated otherwise). Serum markers of immune activation were also measured: soluble CD14, soluble CD163, tumor necrosis factor receptor (TNFR)-I, TNFR-II, vascular cell adhesion molecule (VCAM)-1, intracellular adhesion molecule (ICAM)-1, CX3CL1, C-reactive protein (CRP), myeloperoxidase (MPO), LPS-binding protein (LBP); D-dimer (Stago).

### Measurement of intracellular reactive oxygen species

To measure reactive oxygen species production, MDMs were incubated with 5 μM CellROX Deep Red (Thermo Fisher) at 37C for 30 min, and then analyzed by flow cytometry at ~640/665 nm. For stimulation experiments, MDMs were treated with LPS (200 ng/mL) for 90 min prior to incubation with CellROX reagent.

### MMP activity

General MMP activity in MDM supernatants was measured using the SensoLyte Generic MMP colorimetric assay kit (AnaSpec) following manufacturer’s protocols. A microplate reader was used to measure absorbance at 412 nm.

### Measurements of foam cell formation

To evaluate intracellular lipid accumulation, MDMs were stained with 0.25 ug/mL Bodipy 493/503 (Life Technologies) for 20 min in the dark at room temperature, and then analyzed by flow cytometry. For DiI-OxLDL (Alfa Aesar) uptake experiments, MDMs in Teflon wells were resuspended in RPMI 1640 supplemented with 2% autologous serum, and then incubated with 10 ug/mL DiI-OxLDL for 4 h. Cells were washed twice with PBS, and mean fluorescence intensity (MFI) of DiI staining was measured by flow cytometry.

### Lipid measurement

Serum lipids were analyzed using the direct infusion-tandem mass spectrometry (DI-MS/MS) Lipidyzer platform (Sciex, MA, USA) that identifies and quantifies ~1,100 biological lipids covering 13 lipid classes (i.e. free fatty acids, ceramides, diacylglycerols, triacylglycerols). The Lipidyzer platform methodology has been described in detail elsewhere [99], but briefly, lipids were extracted from 100 μL of serum using a modified Bligh-Dyer method. Over 50 stable isotope labeled internal standards spanning all 13 lipid classes were added to each sample prior to extraction for accurate quantitation. Extracts were reconstituted in dichloromethane/methanol (1:1) and analyzed using DI-MS/MS with DMS separation. A Shimadzu LC system was used for automated infusion of each serum extract and for pumping running and rinse solutions through the lines. Serum extracts were infused into a 5500 QTRAP MS/MS with SelexION DMS technology (Sciex) and lipid species were targeted and quantitated using optimized MS/MS transitions. Data were generated using the Lipidomics Workflow Manager software (Sciex). Results provided the concentration (μM) and fatty acid composition (mol%) of total lipid classes and individual lipid species.

### Phagocytosis of *E*. *coli* bioparticles

MDM phagocytic activity was measured using pHrodo Green *E*. *Coli* bioparticles (Life Technologies). Particles are conjugated to pHrodo dye, which fluoresces in the acidic environment of phagosomes. MDMs were plated in 12-well culture plates (3 X 10^5^ MDMs/mL), and purified by adherence to plastic for 3 h. After 24 h, culture medium was replaced with 500 μl of varying concentrations of pHrodo green *E*. *coli* bioparticles, and cells were incubated for 1 h at 37C. As controls, MDMs were incubated with pHrodo green *E*. *Coli* bioparticles on ice. Cells were washed with PBS and detached by scraping. MDMs were analyzed by flow cytometry and phagocytic activity was determined by measuring fluorescence intensity of cells. Final reported fluorescence intensity was calculated by subtracting intensity of cells incubated at 0C from cells incubated at 37C.

## Supporting information

S1 Fig Surface marker expression differs among monocyte subsets and MDMs from people with and without HIV. Whole blood samples from people with and without HIV were stained for monocyte subset surface markers (CD14 and CD16) and expression of various activation markers was analyzed by flow cytometry. Summary data are shown (* p<0.05, ** p<0.01) (MFI, mean fluorescence intensity).
(TIF)

S2 Fig In vitro CD300e activation and E. coli bioparticle uptake. A) MDMs were cultured for 24h in the presence of anti-CD300e monoclonal antibody (1 μg/mL) or isotype-matched control (IgG1). ROS production was measured by flow cytometry following staining with CellROX deep red. Surface expression of HLA-DR was also assessed by flow cytometry. B) To assess phagocytic capacity of MDMs from donors with and without HIV, cells were exposed for 1 h to increasing amounts of pHrodo-labeled *E*. *coli* bioparticles and analyzed by flow cytometry.
(TIF)

S3 Fig Serum biomarkers of inflammation, and monocyte and endothelial cell activation are increased in PWH. Serum was collected from study participants with and without HIV. Concentrations of markers of inflammation (TNFR1, TNFR2, CRP, IL-6, D-Dimer), endothelial cell activation (ICAM1, VCAM1, CX3CL1), monocyte/macrophage activation (sCD14, sCD163), microbial translocation (LBP), and oxidative stress (MPO) were measured by ELISA. *p<0.05, ** p<0.005.
(TIF)

S4 Fig Biomarkers associated with morbidity and mortality in HIV infection correlate with unique DGE signatures and altered pathway activation. Differentially expressed pathways (DEPs) were identified using gene set variation analysis (GSVA) using the regressed genes selected above from donor serum levels of A) sCD163 and B) TNFR2, and C) ROS production from isolated MDMs and ranked by PCC (P≤0.05).
(TIF)

S5 Fig Lipids associated with inflammation and CVD correlate with unique DGE signatures and altered pathway activation. A) Increased levels of CERs are associated with increased expression of genes associated with the intrinsic and extrinsic coagulation pathways and G-protein receptor signaling and decreased expression of genes associated with lipid metabolism. Differentially expressed pathways (DEPs) were identified using gene set variation analysis (GSVA) and ranked by PCC (P≤0.05).
(TIF)

S6 Fig Proportional representation of monocyte subsets in the blood are associated with differential MDM gene expression. A) Decreased representation of CD14+CD16- traditional monocytes directly ex vivo are associated with increased expression of genes related to lipid processing and cytokine expression in MDMs. B) Increasing representation of CD14+CD16+ inflammatory monocytes in blood are associated with increased expression of genes related to IL-10 signaling and lipid processing and metabolism. Differentially expressed pathways (DEPs) were identified using GSVA and ranked by PCC (P≤0.05).
(TIF)

S7 Fig Pooled serum from PWH are enriched for saturated fatty acids and levels of these lipids are associated with MDM activation. A) The fatty acid composition of total fatty acids is altered in HIV+ pooled serum. B) Concentrations of SaFAs are significantly increased in HIV+ pooled serum. C) Spearman correlations are reported for relationships among FFA species and HLADR expression on MDMs from HIV+ donors. FFAs containing SaFAs (red) were positively associated, and FFAs containing MUFAs (gray) and PUFAs (blue) were inversely associated with HLADR levels. * p<0.05.
(TIF)

S1 Table Gene expression associated with Age. To assess the confounding effect of age in our data, we performed linear regressions to determine its relationship to each gene in the transcriptome. After applying a nominal p-value threshold of 0.05, 287 genes showed a relationship with age.
(DOCX)
