# Supplementary figures and images for: Macrophage maturation from blood monocytes is altered in people with HIV, and is linked to serum lipid profiles and activation indices: A model for studying atherogenic mechanisms
Source: PLoS Pathog. 2020 Oct 1;16(10):e1008869. doi: 10.1371/journal.ppat.1008869 (PMC7553323; doi:10.1371/journal.ppat.1008869)

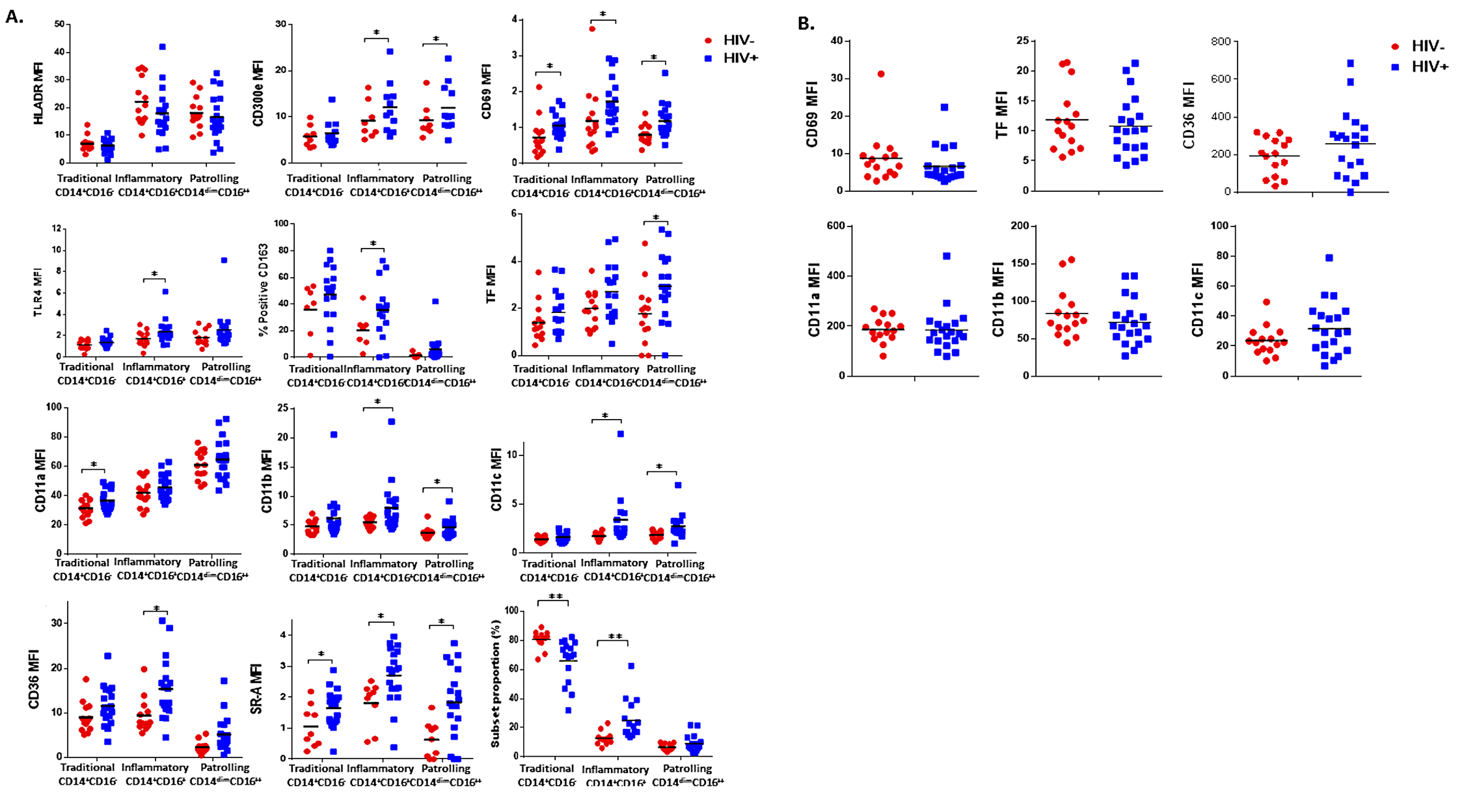

Supplement: S1 Fig — Whole blood samples from people with and without HIV were stained for monocyte subset surface markers (CD14 and CD16) and expression of various activation markers was analyzed by flow cytometry. Summary data are shown (* p<0.05, ** p<0.01) (MFI, mean fluorescence intensity). (TIF) [file ppat.1008869.s001.tif]

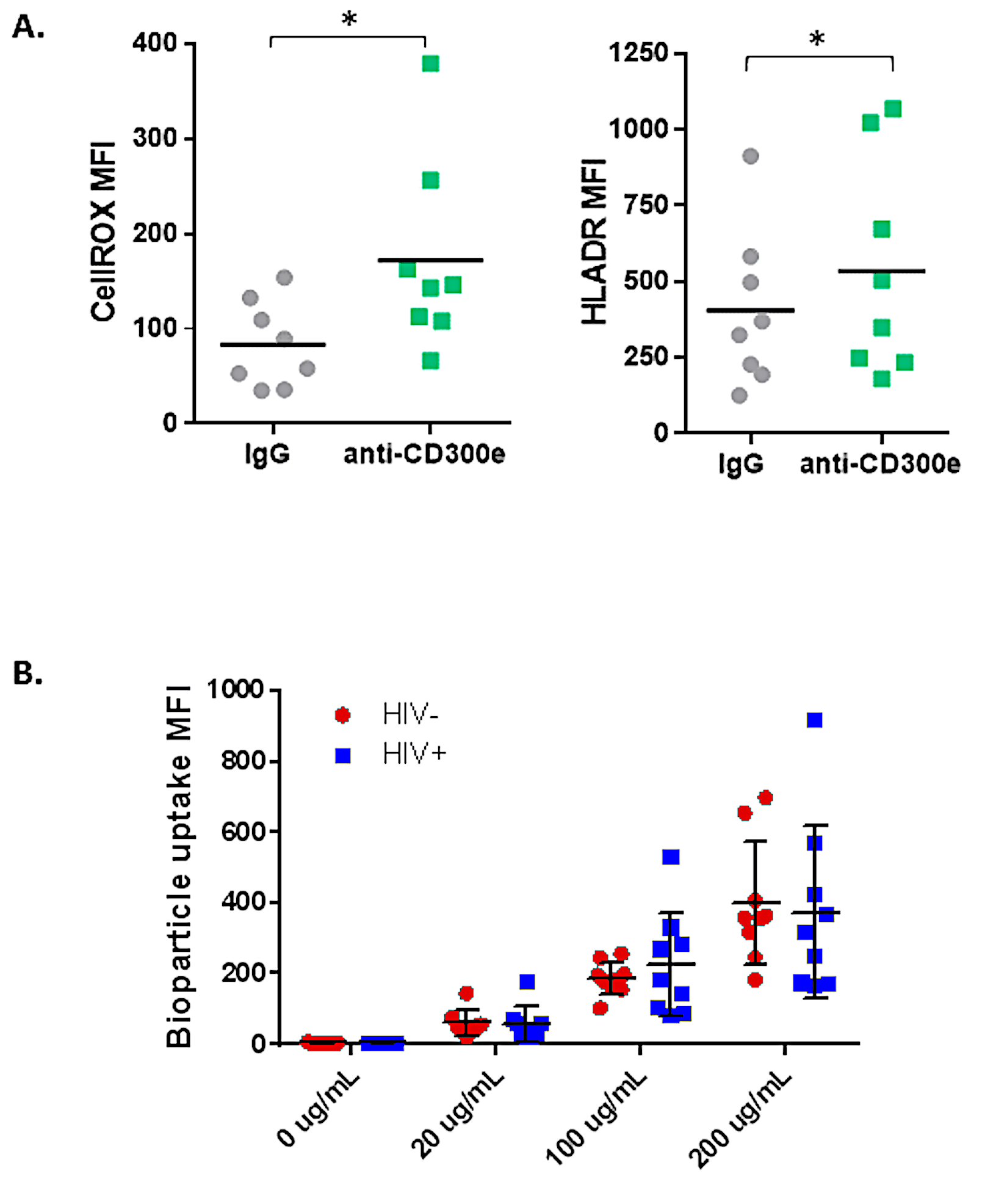

Supplement: S2 Fig — A) MDMs were cultured for 24h in the presence of anti-CD300e monoclonal antibody (1 μg/mL) or isotype-matched control (IgG1). ROS production was measured by flow cytometry following staining with CellROX deep red. Surface expression of HLA-DR was also assessed by flow cytometry. B) To assess phagocytic capacity of MDMs from donors with and without HIV, cells were exposed for 1 h to increasing amounts of pHrodo-labeled E. coli bioparticles and analyzed by flow cytometry. (TIF) [file ppat.1008869.s002.tif]

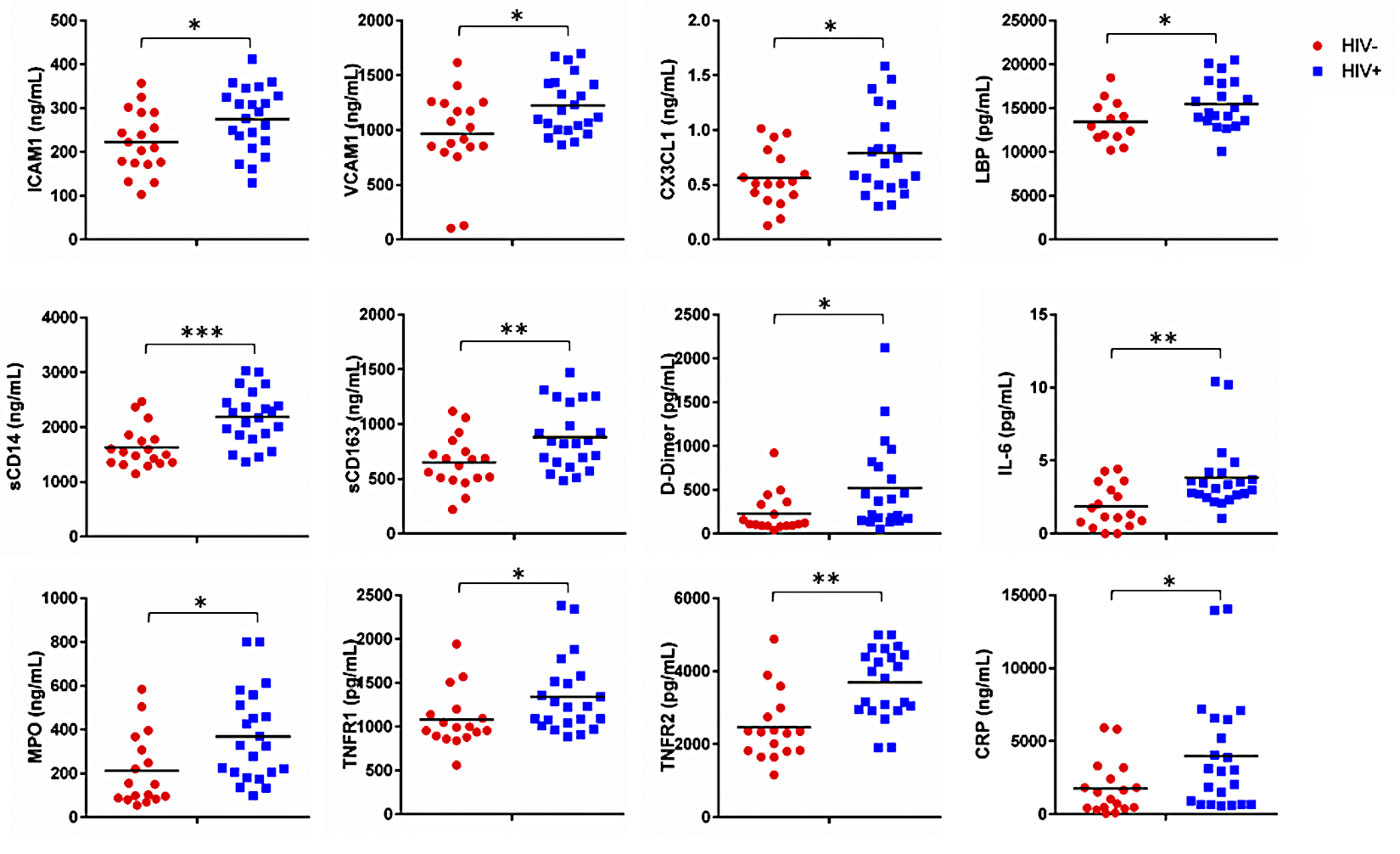

Supplement: S3 Fig — Serum was collected from study participants with and without HIV. Concentrations of markers of inflammation (TNFR1, TNFR2, CRP, IL-6, D-Dimer), endothelial cell activation (ICAM1, VCAM1, CX3CL1), monocyte/macrophage activation (sCD14, sCD163), microbial translocation (LBP), and oxidative stress (MPO) were measured by ELISA. *p<0.05, ** p<0.005. (TIF) [file ppat.1008869.s003.tif]

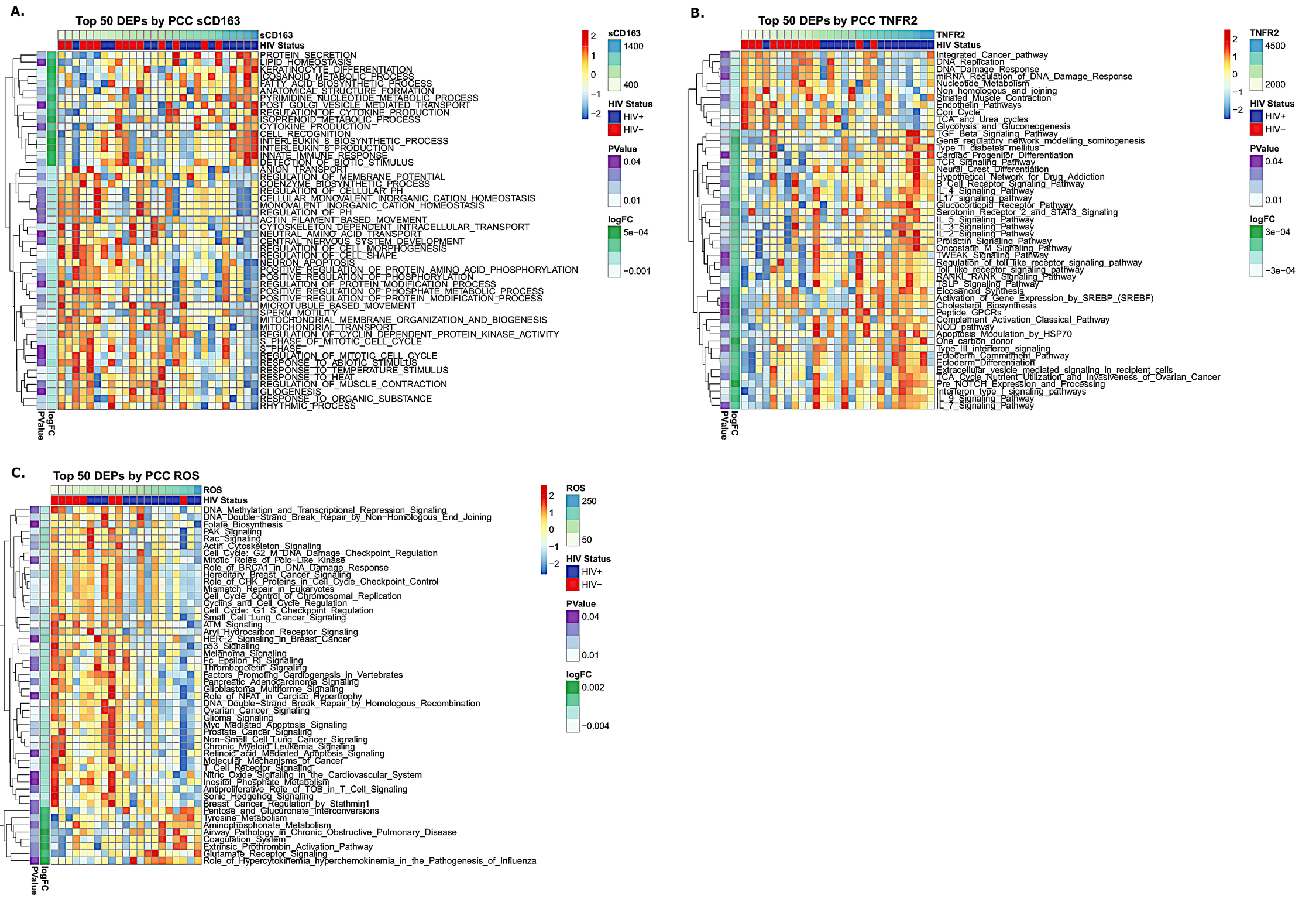

Supplement: S4 Fig — Differentially expressed pathways (DEPs) were identified using gene set variation analysis (GSVA) using the regressed genes selected above from donor serum levels of A) sCD163 and B) TNFR2, and C) ROS production from isolated MDMs and ranked by PCC (P≤0.05). (TIF) [file ppat.1008869.s004.tif]

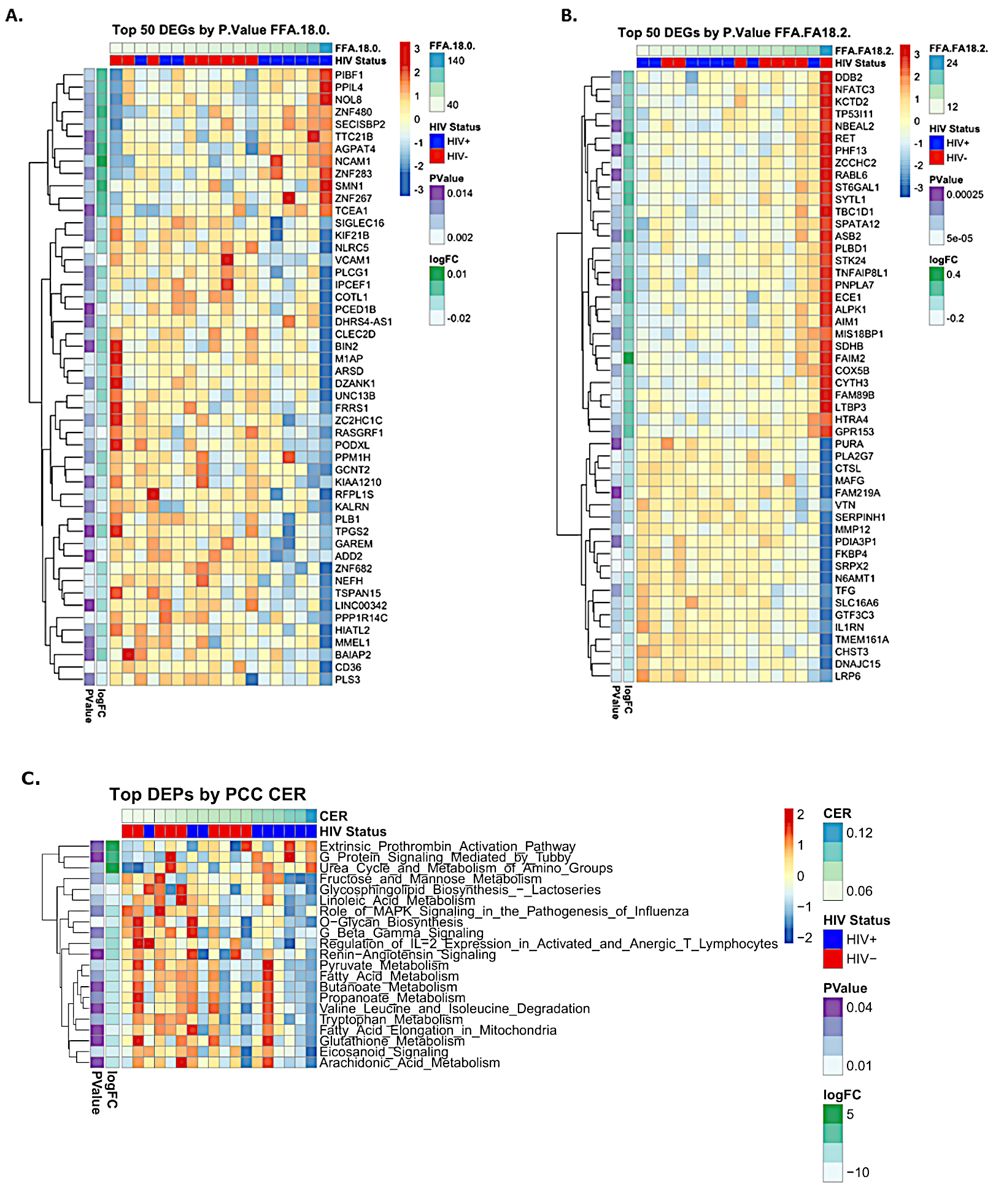

Supplement: S5 Fig — A) Increased levels of CERs are associated with increased expression of genes associated with the intrinsic and extrinsic coagulation pathways and G-protein receptor signaling and decreased expression of genes associated with lipid metabolism. Differentially expressed pathways (DEPs) were identified using gene set variation analysis (GSVA) and ranked by PCC (P≤0.05). (TIF) [file ppat.1008869.s005.tif]

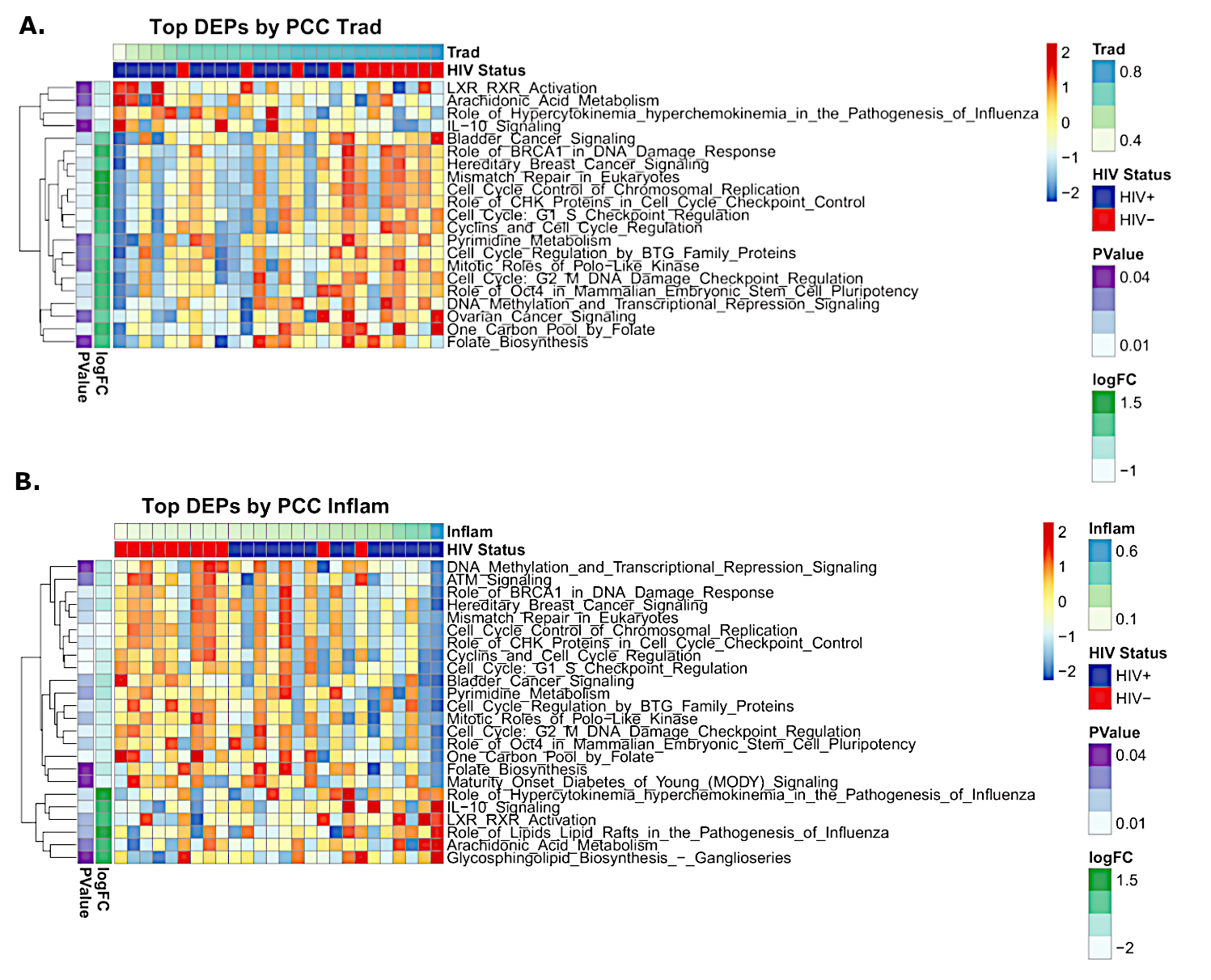

Supplement: S6 Fig — A) Decreased representation of CD14+CD16- traditional monocytes directly ex vivo are associated with increased expression of genes related to lipid processing and cytokine expression in MDMs. B) Increasing representation of CD14+CD16+ inflammatory monocytes in blood are associated with increased expression of genes related to IL-10 signaling and lipid processing and metabolism. Differentially expressed pathways (DEPs) were identified using GSVA and ranked by PCC (P≤0.05). (TIF) [file ppat.1008869.s006.tif]

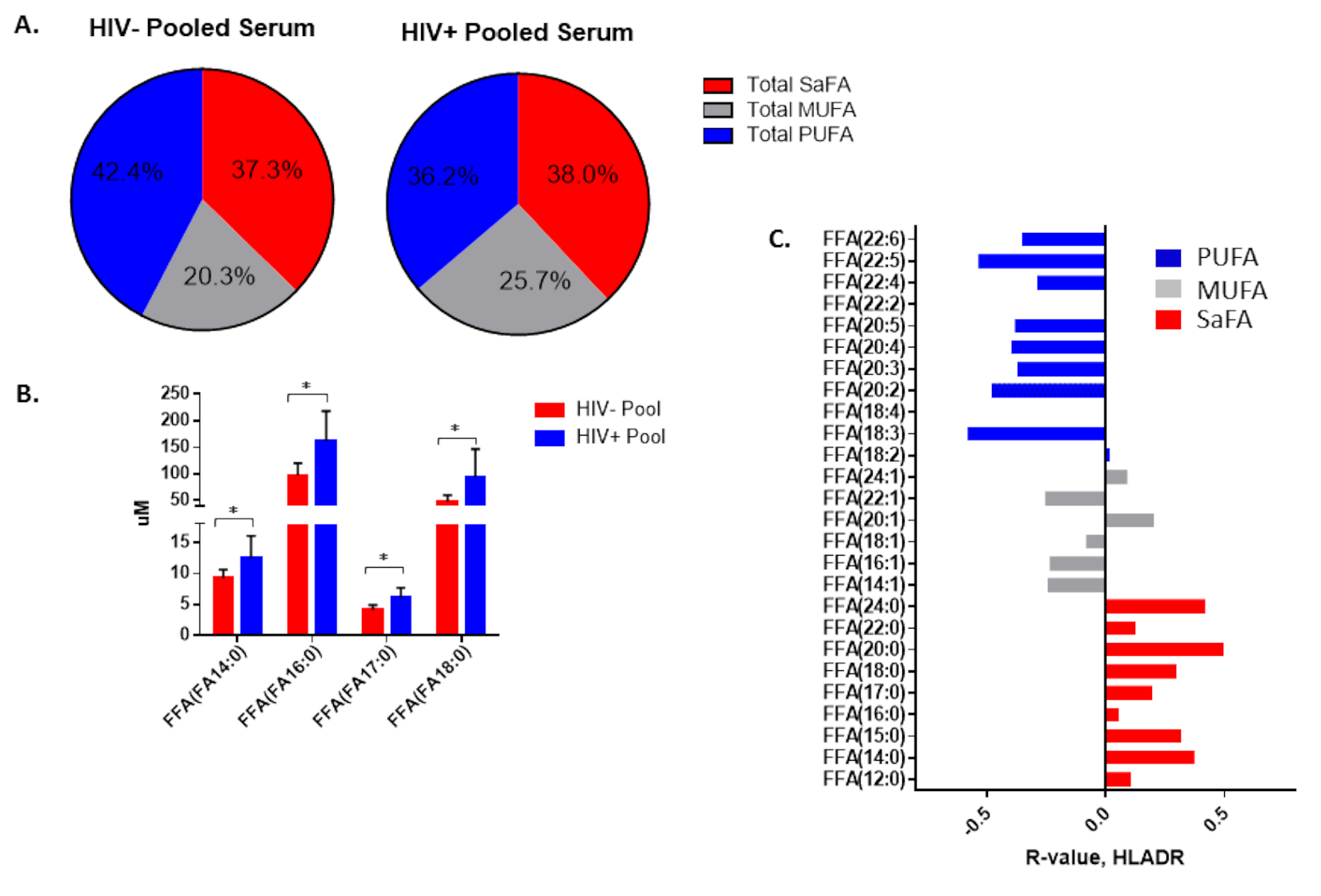

Supplement: S7 Fig — A) The fatty acid composition of total fatty acids is altered in HIV+ pooled serum. B) Concentrations of SaFAs are significantly increased in HIV+ pooled serum. C) Spearman correlations are reported for relationships among FFA species and HLADR expression on MDMs from HIV+ donors. FFAs containing SaFAs (red) were positively associated, and FFAs containing MUFAs (gray) and PUFAs (blue) were inversely associated with HLADR levels. * p<0.05. (TIF) [file ppat.1008869.s007.tif]
