# Supplementary material for: Macrophage maturation from blood monocytes is altered in people with HIV, and is linked to serum lipid profiles and activation indices: A model for studying atherogenic mechanisms
Source: PLoS Pathog. 2020 Oct 1;16(10):e1008869. doi: 10.1371/journal.ppat.1008869 (PMC7553323; doi:10.1371/journal.ppat.1008869)
Supplement: S1 Table — To assess the confounding effect of age in our data, we performed linear regressions to determine its relationship to each gene in the transcriptome. After applying a nominal p-value threshold of 0.05, 287 genes showed a relationship with age. (DOCX) [file ppat.1008869.s008.docx]

| Gene symbol | Age_P.Value | Age_Adj_P.Value | Age_PCC |
| --- | --- | --- | --- |
| ABCB10 | 0.028622053 | 0.99939077 | -0.446799051 |
| ACADVL | 0.037070484 | 0.99939077 | 0.433379194 |
| ACER3 | 0.001451989 | 0.99939077 | -0.606747894 |
| ADD2 | 0.026375699 | 0.99939077 | -0.389764896 |
| ADPRH | 0.023909164 | 0.99939077 | -0.433747013 |
| ADPRM | 0.033076159 | 0.99939077 | -0.487776899 |
| AK8 | 0.046106392 | 0.99939077 | -0.409461418 |
| ALDH3A2 | 0.024560842 | 0.99939077 | -0.434898781 |
| ALG3 | 0.043135481 | 0.99939077 | 0.362269364 |
| AMFR | 0.036031924 | 0.99939077 | -0.393919046 |
| ANPEP | 0.017841443 | 0.99939077 | 0.384315064 |
| APOC1 | 0.044520099 | 0.99939077 | -0.405413307 |
| APOC1P1 | 0.005493696 | 0.99939077 | -0.541210548 |
| APPBP2 | 0.01573495 | 0.99939077 | -0.499047563 |
| ARHGEF12 | 0.044982332 | 0.99939077 | -0.393304548 |
| ARHGEF37 | 0.00559816 | 0.99939077 | -0.494186397 |
| ARL6 | 0.022094153 | 0.99939077 | 0.475833932 |
| ARMCX6 | 0.019685489 | 0.99939077 | 0.49581511 |
| ARNTL | 0.018933214 | 0.99939077 | -0.419182006 |
| ASPM | 0.043489113 | 0.99939077 | -0.373198964 |
| ATP6V1F | 0.039020956 | 0.99939077 | 0.447487471 |
| ATXN7L2 | 0.019935176 | 0.99939077 | 0.495832329 |
| B3GALT6 | 0.043413227 | 0.99939077 | 0.412389086 |
| B4GALT7 | 0.034869465 | 0.99939077 | 0.29882483 |
| BANP | 0.011875238 | 0.99939077 | 0.472726576 |
| BIRC5 | 0.044961989 | 0.99939077 | -0.37616594 |
| BNIP3 | 0.007059409 | 0.99939077 | 0.411509531 |
| BRCA2 | 0.02746377 | 0.99939077 | -0.360224402 |
| BTG1 | 0.024392035 | 0.99939077 | 0.395958611 |
| C11orf49 | 0.043941116 | 0.99939077 | 0.316120078 |
| C1orf53 | 0.030611834 | 0.99939077 | 0.317952127 |
| C20orf26 | 0.025169361 | 0.99939077 | -0.429477491 |
| CARD11 | 0.036243077 | 0.99939077 | -0.414592153 |
| CARD8 | 0.037224583 | 0.99939077 | 0.440888399 |
| CCDC124 | 0.030475245 | 0.99939077 | 0.454806961 |
| CCDC126 | 0.011443201 | 0.99939077 | 0.483852362 |
| CCNA2 | 0.040340271 | 0.99939077 | -0.380761894 |
| CD274 | 0.030497699 | 0.99939077 | -0.373340423 |
| CD27-AS1 | 0.0304131 | 0.99939077 | 0.411645515 |
| CDC20 | 0.049162389 | 0.99939077 | -0.378964382 |
| CDC42EP1 | 0.002297948 | 0.99939077 | -0.561658427 |
| CDCA2 | 0.013863122 | 0.99939077 | -0.449190717 |
| CDCA8 | 0.043234686 | 0.99939077 | -0.387759602 |
| CDK6 | 0.042978931 | 0.99939077 | -0.3974436 |
| CDKN3 | 0.027935965 | 0.99939077 | -0.41740946 |
| CENPA | 0.042194412 | 0.99939077 | -0.388352992 |
| CENPF | 0.049490883 | 0.99939077 | -0.360086387 |
| CEP55 | 0.04193578 | 0.99939077 | -0.376436066 |
| CHTOP | 0.037846085 | 0.99939077 | 0.404110017 |
| CLEC11A | 0.041587266 | 0.99939077 | 0.418834441 |
| CLN8 | 0.02125984 | 0.99939077 | 0.441738753 |
| CLTB | 0.01769162 | 0.99939077 | 0.440187996 |
| CMTM6 | 0.039171057 | 0.99939077 | -0.410215312 |
| COIL | 0.047455515 | 0.99939077 | 0.471957628 |
| COL4A2-AS2 | 0.018305777 | 0.99939077 | -0.451740415 |
| COPZ2 | 0.025080764 | 0.99939077 | -0.436641288 |
| CXXC1 | 0.01644165 | 0.99939077 | 0.491025798 |
| CYB5R2 | 0.046026703 | 0.99939077 | -0.352518953 |
| DCUN1D1 | 0.035353663 | 0.99939077 | -0.461922266 |
| DCXR | 0.038269398 | 0.99939077 | 0.393727491 |
| DEPDC1 | 0.019899039 | 0.99939077 | -0.422489523 |
| DHPS | 0.024304001 | 0.99939077 | 0.467585285 |
| DLGAP5 | 0.048528315 | 0.99939077 | -0.368397841 |
| DNMT3A | 0.048212023 | 0.99939077 | -0.341776353 |
| EFCAB2 | 0.021442889 | 0.99939077 | 0.434060843 |
| EFNA4 | 0.018073619 | 0.99939077 | 0.475041628 |
| EIF2AK4 | 0.021428487 | 0.99939077 | -0.427240182 |
| EIF4E3 | 0.029459846 | 0.99939077 | -0.385486903 |
| ELOVL1 | 0.045571751 | 0.99939077 | 0.342809804 |
| EMR4P | 0.046731965 | 0.99939077 | 0.391245525 |
| ENTPD4 | 0.049864395 | 0.99939077 | 0.376887879 |
| ERV3-1 | 0.040235708 | 0.99939077 | 0.42109791 |
| EVC | 0.007802686 | 0.99939077 | 0.464406031 |
| EVC2 | 0.017106475 | 0.99939077 | 0.392765897 |
| FAM129A | 0.000312531 | 0.99939077 | -0.589161573 |
| FAM173A | 0.015129398 | 0.99939077 | 0.419952101 |
| FAM64A | 0.028312552 | 0.99939077 | -0.427284998 |
| FAM83D | 0.047622646 | 0.99939077 | -0.384729562 |
| FCGR1A | 0.01304596 | 0.99939077 | -0.465180661 |
| FCGR3B | 0.016725458 | 0.99939077 | -0.447882221 |
| FGD6 | 0.02287364 | 0.99939077 | -0.288648725 |
| FHAD1 | 0.01837658 | 0.99939077 | -0.436932927 |
| FKBP4 | 0.038272349 | 0.99939077 | 0.419782301 |
| GCLM | 0.041887828 | 0.99939077 | -0.404567518 |
| GDAP1 | 0.047004847 | 0.99939077 | -0.389273906 |
| GGH | 0.012659111 | 0.99939077 | -0.49189733 |
| GORAB | 0.045284899 | 0.99939077 | 0.431266036 |
| GSR | 0.026462745 | 0.99939077 | -0.423387904 |
| GTF3A | 0.023240136 | 0.99939077 | 0.463419167 |
| GTSE1 | 0.047707557 | 0.99939077 | -0.361169409 |
| H3F3C | 0.042277055 | 0.99939077 | 0.350034668 |
| HIVEP2 | 0.020537953 | 0.99939077 | -0.432070027 |
| HMMR | 0.031775985 | 0.99939077 | -0.397753118 |
| HSD11B1 | 0.043793909 | 0.99939077 | -0.40300529 |
| ICAM5 | 0.019875367 | 0.99939077 | 0.432451401 |
| IFITM10 | 0.035624207 | 0.99939077 | -0.383776406 |
| IL1RN | 0.03563395 | 0.99939077 | 0.405906535 |
| INPP1 | 0.024659397 | 0.99939077 | -0.463021791 |
| INTU | 0.001747815 | 0.99939077 | 0.604415128 |
| ITGA2 | 0.040473554 | 0.99939077 | 0.40427196 |
| JAKMIP2 | 0.042714425 | 0.99939077 | 0.367482807 |
| JMJD8 | 0.010502833 | 0.99939077 | 0.525701267 |
| JOSD2 | 0.048760162 | 0.99939077 | 0.218823639 |
| KCTD20 | 0.023153116 | 0.99939077 | -0.495389958 |
| KCTD9 | 0.009334508 | 0.99939077 | -0.439678242 |
| KHDRBS3 | 0.040298823 | 0.99939077 | -0.317075792 |
| KIAA1107 | 0.043670118 | 0.99939077 | 0.447609447 |
| KIAA1324L | 0.028552514 | 0.99939077 | -0.421939611 |
| KIAA1524 | 0.037817712 | 0.99939077 | -0.388792129 |
| KIF11 | 0.048751895 | 0.99939077 | -0.357351449 |
| KIF14 | 0.049332988 | 0.99939077 | -0.373459568 |
| KIF20A | 0.032567284 | 0.99939077 | -0.392537225 |
| KIF23 | 0.034188373 | 0.99939077 | -0.391490567 |
| KIFC3 | 0.003188813 | 0.99939077 | 0.432050798 |
| KLRB1 | 0.04177109 | 0.99939077 | -0.395425852 |
| KPNA2 | 0.042586027 | 0.99939077 | -0.38111972 |
| LAMTOR1 | 0.04674207 | 0.99939077 | 0.140348552 |
| LGALS2 | 0.010054025 | 0.99939077 | -0.487718987 |
| LIN37 | 0.0334059 | 0.99939077 | 0.46921653 |
| LIN7B | 0.017011361 | 0.99939077 | 0.458461722 |
| LINC00338 | 0.023524451 | 0.99939077 | -0.497438534 |
| LINC00843 | 0.010824613 | 0.99939077 | -0.525780636 |
| LINC00969 | 0.041496221 | 0.99939077 | 0.394278057 |
| LINC01004 | 0.021186588 | 0.99939077 | 0.472927613 |
| LITAF | 0.03047688 | 0.99939077 | -0.348655837 |
| LOC100093631 | 0.012529596 | 0.99939077 | -0.449109389 |
| LOC100506990 | 0.043217727 | 0.99939077 | -0.383253768 |
| LOC101927069 | 0.013583429 | 0.99939077 | -0.487544089 |
| LOC101927151 | 0.012869512 | 0.99939077 | 0.516877687 |
| LOC101927663 | 0.040459531 | 0.99939077 | 0.416815422 |
| LOC101928228 | 0.025532583 | 0.99939077 | -0.40198248 |
| LOC101929758 | 0.012953968 | 0.99939077 | -0.527250915 |
| LOC102724050 | 0.016406018 | 0.99939077 | 0.358725135 |
| LOC728026 | 0.03717481 | 0.99939077 | 0.398078331 |
| LRRC37A2 | 0.040539113 | 0.99939077 | 0.378884441 |
| LRRC8D | 0.004854583 | 0.99939077 | -0.539326416 |
| LRRFIP2 | 0.044814048 | 0.99939077 | -0.294704084 |
| LRRK1 | 0.018779867 | 0.99939077 | -0.462491108 |
| LYPLAL1 | 0.015852902 | 0.99939077 | -0.494123925 |
| MALT1 | 0.019827588 | 0.99939077 | -0.388283762 |
| MANEA | 0.018515101 | 0.99939077 | 0.396853181 |
| MARCH5 | 0.007781079 | 0.99939077 | -0.537801479 |
| MCM9 | 0.026559844 | 0.99939077 | -0.467637507 |
| ME1 | 0.012400848 | 0.99939077 | -0.484606177 |
| MED19 | 0.036068363 | 0.99939077 | 0.412260671 |
| METRN | 0.025846419 | 0.99939077 | 0.420671125 |
| METTL4 | 0.048106384 | 0.99939077 | -0.419427281 |
| MFSD10 | 0.036398838 | 0.99939077 | 0.135034299 |
| MICB | 0.042554982 | 0.99939077 | -0.305748235 |
| MKI67 | 0.045532919 | 0.99939077 | -0.338045867 |
| MOB3B | 0.017674878 | 0.99939077 | -0.387555361 |
| MRC2 | 0.011035457 | 0.99939077 | -0.409035545 |
| MSL3P1 | 0.020526598 | 0.99939077 | -0.432194642 |
| MT1F | 0.010649091 | 0.99939077 | 0.456233284 |
| MT1G | 0.016625821 | 0.99939077 | 0.441736322 |
| MT1H | 0.039037332 | 0.99939077 | 0.396256031 |
| MT1X | 0.005996918 | 0.99939077 | 0.471765448 |
| MT2A | 0.03393836 | 0.99939077 | 0.404942671 |
| MTMR14 | 0.017311176 | 0.99939077 | -0.495827602 |
| MTO1 | 0.007269412 | 0.99939077 | -0.486531928 |
| NAPSB | 0.024866674 | 0.99939077 | -0.432338056 |
| NCAPG2 | 0.032944783 | 0.99939077 | -0.392521001 |
| NDC80 | 0.043876352 | 0.99939077 | -0.377808994 |
| NDUFAF6 | 0.022926886 | 0.99939077 | -0.441697961 |
| NEFH | 0.006100936 | 0.99939077 | -0.455163334 |
| NEK2 | 0.044946171 | 0.99939077 | -0.358635375 |
| NUBP2 | 0.018172359 | 0.99939077 | 0.401786542 |
| NUDT16L1 | 0.030075675 | 0.99939077 | 0.267996211 |
| OLFML2B | 0.036587603 | 0.99939077 | 0.362582086 |
| ORAI2 | 0.043386949 | 0.99939077 | -0.424060747 |
| OTUB1 | 0.042196037 | 0.99939077 | 0.329590439 |
| PALLD | 0.029708013 | 0.99939077 | -0.331406355 |
| PANK4 | 0.031648284 | 0.99939077 | 0.377570959 |
| PARPBP | 0.047211082 | 0.99939077 | -0.402117901 |
| PITPNA-AS1 | 0.027062374 | 0.99939077 | 0.463751991 |
| PLCL1 | 0.037017299 | 0.99939077 | -0.401957862 |
| PLEK2 | 0.047140553 | 0.99939077 | -0.382039183 |
| PLK1 | 0.020194619 | 0.99939077 | -0.439645555 |
| PMS2P4 | 0.047727763 | 0.99939077 | -0.437068862 |
| POLE4 | 0.016198197 | 0.99939077 | 0.446471916 |
| POLR2F | 0.03594221 | 0.99939077 | 0.410747297 |
| POLR2L | 0.013835116 | 0.99939077 | 0.383232597 |
| PPP1R3D | 0.046564901 | 0.99939077 | 0.437090486 |
| PRRG1 | 0.017296699 | 0.99939077 | 0.413611631 |
| PSEN2 | 0.036153071 | 0.99939077 | -0.326453629 |
| PTCRA | 0.032998428 | 0.99939077 | -0.431969657 |
| PTMA | 0.031500617 | 0.99939077 | 0.429990016 |
| PTPRM | 0.007375314 | 0.99939077 | 0.514811277 |
| RAB13 | 0.030809634 | 0.99939077 | 0.427732451 |
| RAB18 | 0.030933045 | 0.99939077 | -0.427067416 |
| RABEP2 | 0.006582034 | 0.99939077 | 0.44652171 |
| RALGAPA1 | 0.049950998 | 0.99939077 | 0.434197319 |
| RBBP8 | 0.047106277 | 0.99939077 | -0.305337099 |
| RBM23 | 0.040895496 | 0.99939077 | 0.360544332 |
| RC3H1 | 0.035936493 | 0.99939077 | -0.283903104 |
| RFPL1S | 0.003290832 | 0.99939077 | -0.551060101 |
| RFX2 | 0.026765345 | 0.99939077 | 0.415556635 |
| RFXANK | 0.019582943 | 0.99939077 | 0.439474631 |
| RNU11 | 0.016381988 | 0.99939077 | -0.412613008 |
| RNVU1-14 | 0.026174787 | 0.99939077 | -0.456837134 |
| RP11-695J4.2 | 0.021000522 | 0.99939077 | -0.469783092 |
| RP11-701P16.5 | 0.024301934 | 0.99939077 | -0.445084427 |
| RP4-756H11.3 | 0.0414819 | 0.99939077 | -0.352676589 |
| RPGRIP1 | 0.0366018 | 0.99939077 | -0.42082528 |
| RPS15 | 0.025732448 | 0.99939077 | 0.466260429 |
| SCARNA2 | 0.047029698 | 0.99939077 | 0.12976533 |
| SEPT9 | 0.019444824 | 0.99939077 | -0.426235573 |
| SH3BGRL3 | 0.030388315 | 0.99939077 | 0.45161417 |
| SHC4 | 0.030839526 | 0.99939077 | 0.35323228 |
| SIPA1L2 | 0.024323838 | 0.99939077 | -0.41782089 |
| SKA1 | 0.034768526 | 0.99939077 | -0.389214122 |
| SLC23A2 | 0.009140784 | 0.99939077 | -0.512854825 |
| SLC25A12 | 0.008264536 | 0.99939077 | -0.519884933 |
| SLC25A44 | 0.036317015 | 0.99939077 | -0.458978653 |
| SLC41A2 | 0.045517402 | 0.99939077 | -0.380638515 |
| SLC47A1 | 0.028254354 | 0.99939077 | -0.413309451 |
| SLC48A1 | 0.016589334 | 0.99939077 | -0.453211253 |
| SNORD3B-1 | 0.009265319 | 0.99939077 | 0.403854537 |
| SNTB2 | 0.045447718 | 0.99939077 | 0.294282343 |
| SPATA20 | 0.034102774 | 0.99939077 | 0.252777419 |
| SPATC1L | 0.036827155 | 0.99939077 | 0.373508962 |
| SPICE1 | 0.024574368 | 0.99939077 | 0.504917172 |
| SPIRE1 | 0.032865647 | 0.99939077 | -0.328094518 |
| SPON2 | 0.031128127 | 0.99939077 | -0.430935039 |
| SPRYD4 | 0.021416978 | 0.99939077 | -0.48754444 |
| SPTBN5 | 0.032870216 | 0.99939077 | -0.391743011 |
| SRGAP2D | 0.009409967 | 0.99939077 | 0.478034951 |
| SRR | 0.023417438 | 0.99939077 | 0.435128869 |
| SSNA1 | 0.049634689 | 0.99939077 | 0.321951818 |
| SUCNR1 | 0.041938722 | 0.99939077 | -0.378435176 |
| SUDS3 | 0.007401297 | 0.99939077 | 0.545562192 |
| SVIL | 0.044933532 | 0.99939077 | -0.350257861 |
| SYNGAP1 | 0.028805052 | 0.99939077 | 0.433232789 |
| TACC3 | 0.030526515 | 0.99939077 | -0.410132148 |
| TAF11 | 0.037351779 | 0.99939077 | -0.455225954 |
| TATDN3 | 0.030394968 | 0.99939077 | -0.46040072 |
| TBC1D1 | 0.048481879 | 0.99939077 | -0.336079207 |
| TCEANC | 0.036531284 | 0.99939077 | 0.456783523 |
| TFDP1 | 0.026966428 | 0.99939077 | -0.408486988 |
| THAP2 | 0.024339 | 0.99939077 | 0.485289861 |
| TIFA | 0.034472451 | 0.99939077 | -0.413654505 |
| TLE1 | 0.03315195 | 0.99939077 | 0.385408013 |
| TMED4 | 0.011419573 | 0.99939077 | 0.438874758 |
| TMEM106C | 0.0319635 | 0.99939077 | -0.418019137 |
| TMEM134 | 0.015799501 | 0.99939077 | 0.498126418 |
| TMEM154 | 0.020409782 | 0.99939077 | -0.447404542 |
| TMEM229B | 0.012165164 | 0.99939077 | -0.475573804 |
| TMEM231 | 0.021796696 | 0.99939077 | 0.416624119 |
| TMEM234 | 0.047173309 | 0.99939077 | 0.358553742 |
| TMEM245 | 0.045914006 | 0.99939077 | -0.423666054 |
| TMEM258 | 0.012374703 | 0.99939077 | 0.288387763 |
| TMEM67 | 0.027331907 | 0.99939077 | 0.412032178 |
| TOP2A | 0.037271705 | 0.99939077 | -0.377316465 |
| TOX4 | 0.04715274 | 0.99939077 | -0.473990923 |
| TPBG | 0.001244685 | 0.99939077 | 0.583480641 |
| TRNP | 0.01016876 | 0.99939077 | 0.52892639 |
| TRNW | 0.018324863 | 0.99939077 | 0.493061907 |
| TSKU | 0.019168163 | 0.99939077 | -0.463688125 |
| TSPYL5 | 0.005598648 | 0.99939077 | -0.528362457 |
| TTC27 | 0.021793905 | 0.99939077 | 0.398041181 |
| TTC38 | 0.02671816 | 0.99939077 | -0.377396436 |
| TUSC1 | 0.028022887 | 0.99939077 | 0.41856426 |
| TXLNG | 0.043346522 | 0.99939077 | 0.444498708 |
| TXNDC15 | 0.042820191 | 0.99939077 | 0.375356111 |
| UBXN11 | 0.00064218 | 0.99939077 | 0.62270722 |
| UQCC2 | 0.026547205 | 0.99939077 | 0.361489297 |
| USP4 | 0.033107102 | 0.99939077 | 0.381721381 |
| USP8 | 0.034831326 | 0.99939077 | -0.32568478 |
| WAC-AS1 | 0.001984942 | 0.99939077 | -0.565910972 |
| WARS2 | 0.014376763 | 0.99939077 | 0.492460123 |
| WTH3DI | 0.00597503 | 0.99939077 | 0.532123227 |
| ZBED5 | 0.034650534 | 0.99939077 | 0.463959919 |
| ZBTB7B | 0.03105588 | 0.99939077 | 0.395157084 |
| ZC3H12C | 0.022165422 | 0.99939077 | -0.447027066 |
| ZFHX2 | 0.006796671 | 0.99939077 | 0.554842 |
| ZFP62 | 0.018782759 | 0.99939077 | 0.487168378 |
| ZFP91 | 0.038801852 | 0.99939077 | -0.268894231 |
| ZG16B | 0.02604522 | 0.99939077 | -0.399122802 |
| ZNF19 | 0.034751067 | 0.99939077 | 0.450823158 |
| ZNF226 | 0.024037364 | 0.99939077 | 0.489169869 |
| ZNF322 | 0.006666809 | 0.99939077 | 0.537338151 |
| ZNF518B | 0.021508478 | 0.99939077 | -0.40518918 |
| ZNF618 | 0.023712312 | 0.99939077 | -0.395219875 |
| ZNF627 | 0.020041579 | 0.99939077 | 0.419773018 |
| ZNF667 | 0.030973222 | 0.99939077 | 0.437072604 |
| ZNF780A | 0.010306703 | 0.99939077 | 0.498349672 |
| ZNHIT1 | 0.013878493 | 0.99939077 | 0.39653699 |

**Supplementary Table 1. Gene expression associated with age.** To assess the confounding effect of age in our data, we performed linear regressions to determine its relationship to each gene in the transcriptome. After applying a nominal p-value threshold of 0.05, 287 genes showed a relationship with age.
